# Supplementary figures and images for: TXNIP loss expands Myc-dependent transcriptional programs by increasing Myc genomic binding
Source: PLoS Biol. 2023 Mar 17;21(3):e3001778. doi: 10.1371/journal.pbio.3001778 (PMC10058090; doi:10.1371/journal.pbio.3001778)

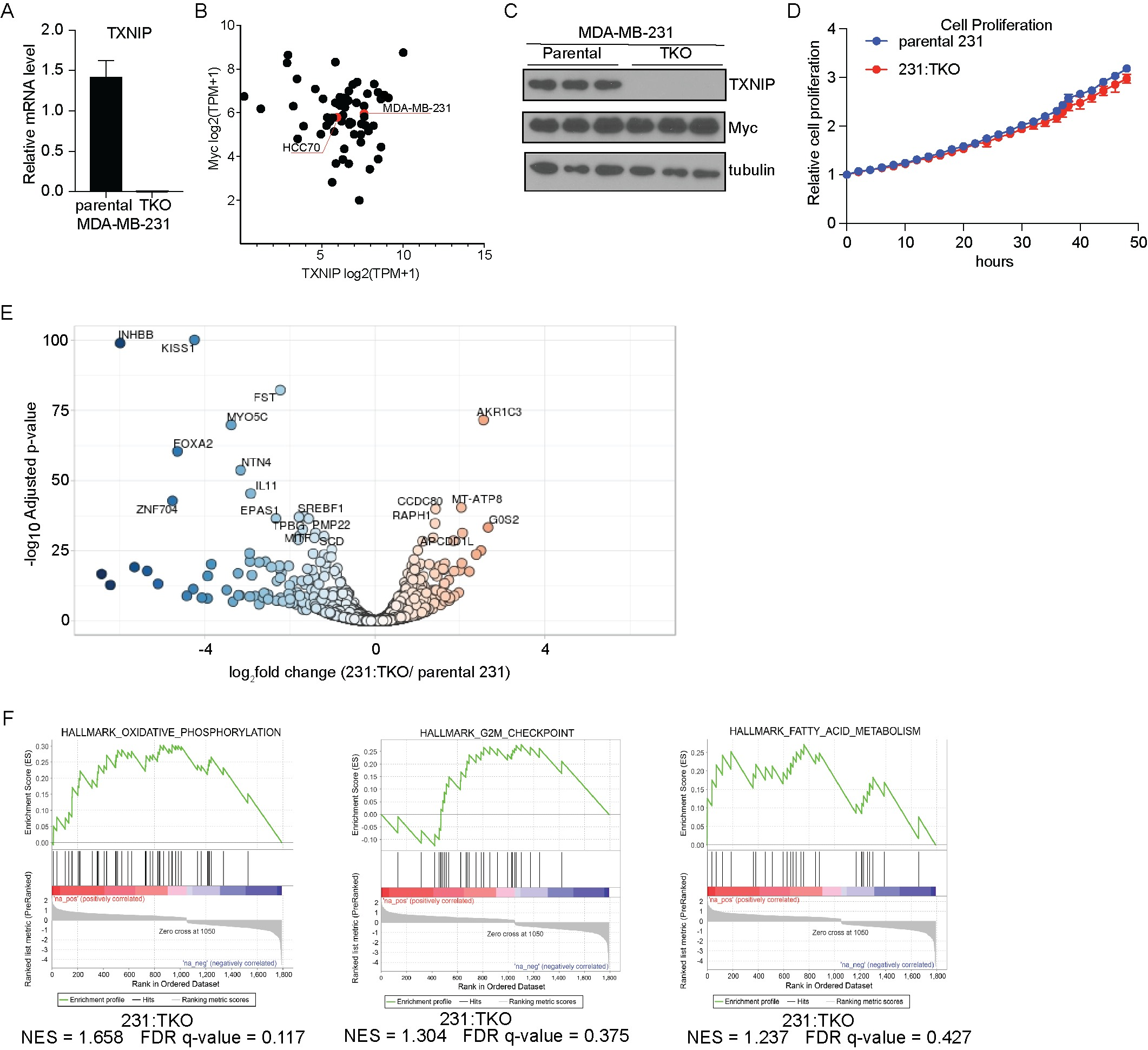

Supplement: S1 Fig — Related to Fig 1. (A) The relative TXNIP mRNA levels (normalized to that of β-actin) in parental 231 and 231:TKO cells were determined by RT-qPCR. (B) The expression levels of Myc and TXNIP from approximately 70 breast cancer cells were plotted. Their expression in HCC70 and MDA-MB-231 cells is indicated. (C) Western blotting was used to determine the levels of Myc, TXNIP, and tubulin in 3 biological replicates of parental 231 and 231:TKO cells. (D) Cell proliferation for parental 231 and 231:TKO cells in regular medium over a 48-hour time course was measured based on the percentage of confluency using real-time videography. (E) A volcano plot of the fold changes and adjusted p-values of regulated transcripts in 231:TKO cells. Gene expression changes in 231:TKO cells were determined using DESeq2. (F) A preranked GSEA enrichment plots of the regulated transcripts in 231:TKO cells with the indicated Hallmark datasets. The underlying data for S1A, S1B, S1D and S1E Fig can be found in S1 Data. GSEA, Gene Set Enrichment Analysis; RT-qPCR, reverse transcription-quantitative PCR; TKO, TXNIP-knockout; TXNIP, thioredoxin interacting protein. (TIF) [file pbio.3001778.s001.tif]

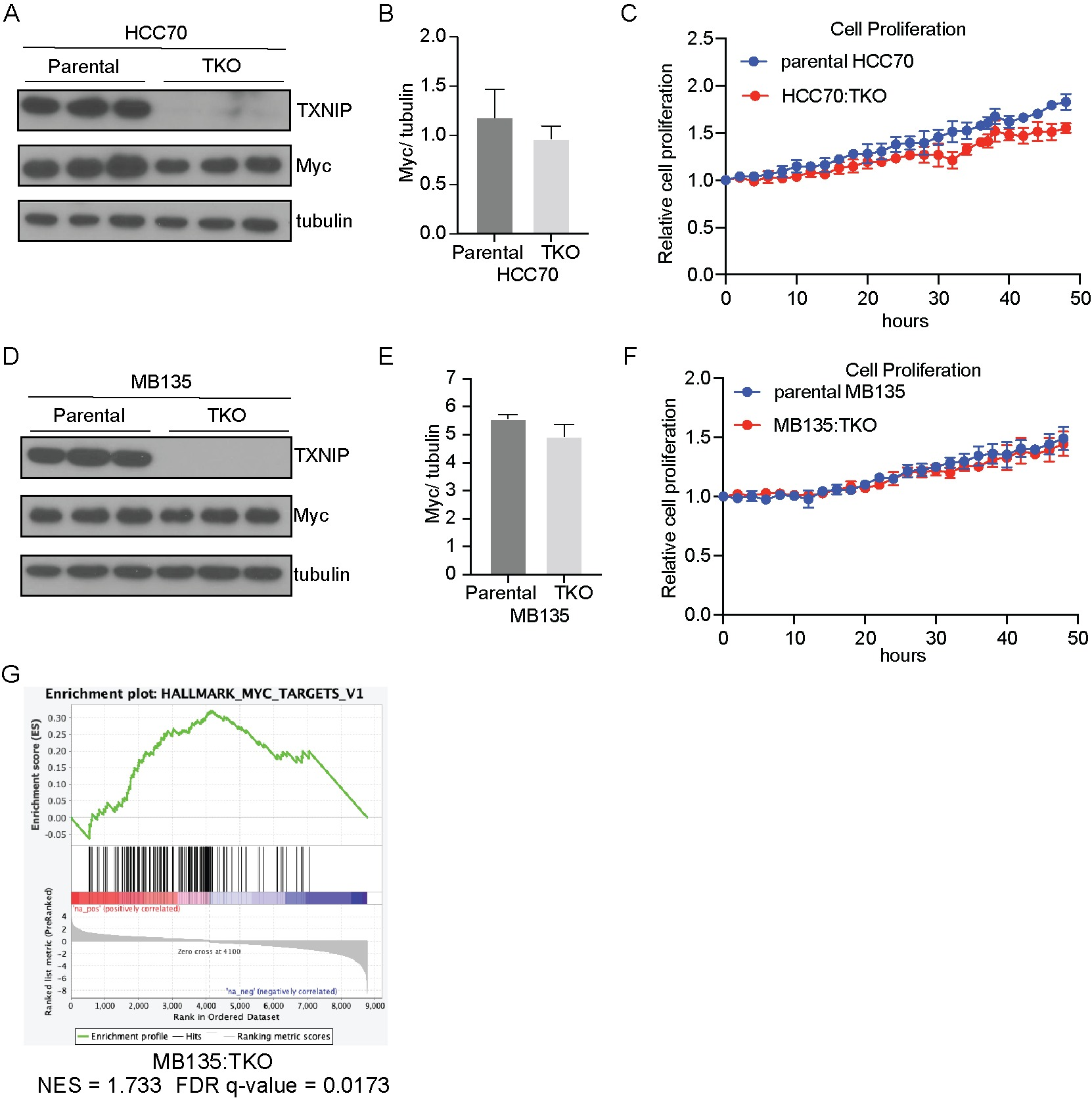

Supplement: S2 Fig — Related to Fig 1. (A) Western blotting was used to determine the levels of Myc, TXNIP, and tubulin in 3 biological replicates of parental HCC70 and HCC70:TKO cells. (B) The levels of Myc protein from (A) in parental HCC70 and HCC70:TKO were quantified using ImageJ. (C) Cell proliferation for parental HCC70 and HCC70:TKO cells in regular medium over a 48-hour time course was measured based on the percentage of confluency using real-time videography. (D) Western blotting was used to determine the levels of Myc, TXNIP, and tubulin in parental MB135 and MB135:TKO cells. (E) The levels of Myc protein from (D) in parental MB135 and 135:TKO were quantified using ImageJ. (F) Cell proliferation for parental MB135 and MB135:TKO cells in regular medium over a 48-hour time course was measured based on the percentage of confluency using real-time videography. (G) A preranked GSEA enrichment plot of regulated transcripts in differentiated myoblast MB135:TKO cells with the Hallmark_Myc_Targets_V1 dataset. The underlying data for S2B, S2C, S2E and S2F Fig can be found in S1 Data. GSEA, Gene Set Enrichment Analysis; TKO, TXNIP-knockout; TXNIP, thioredoxin interacting protein. (TIF) [file pbio.3001778.s002.tif]

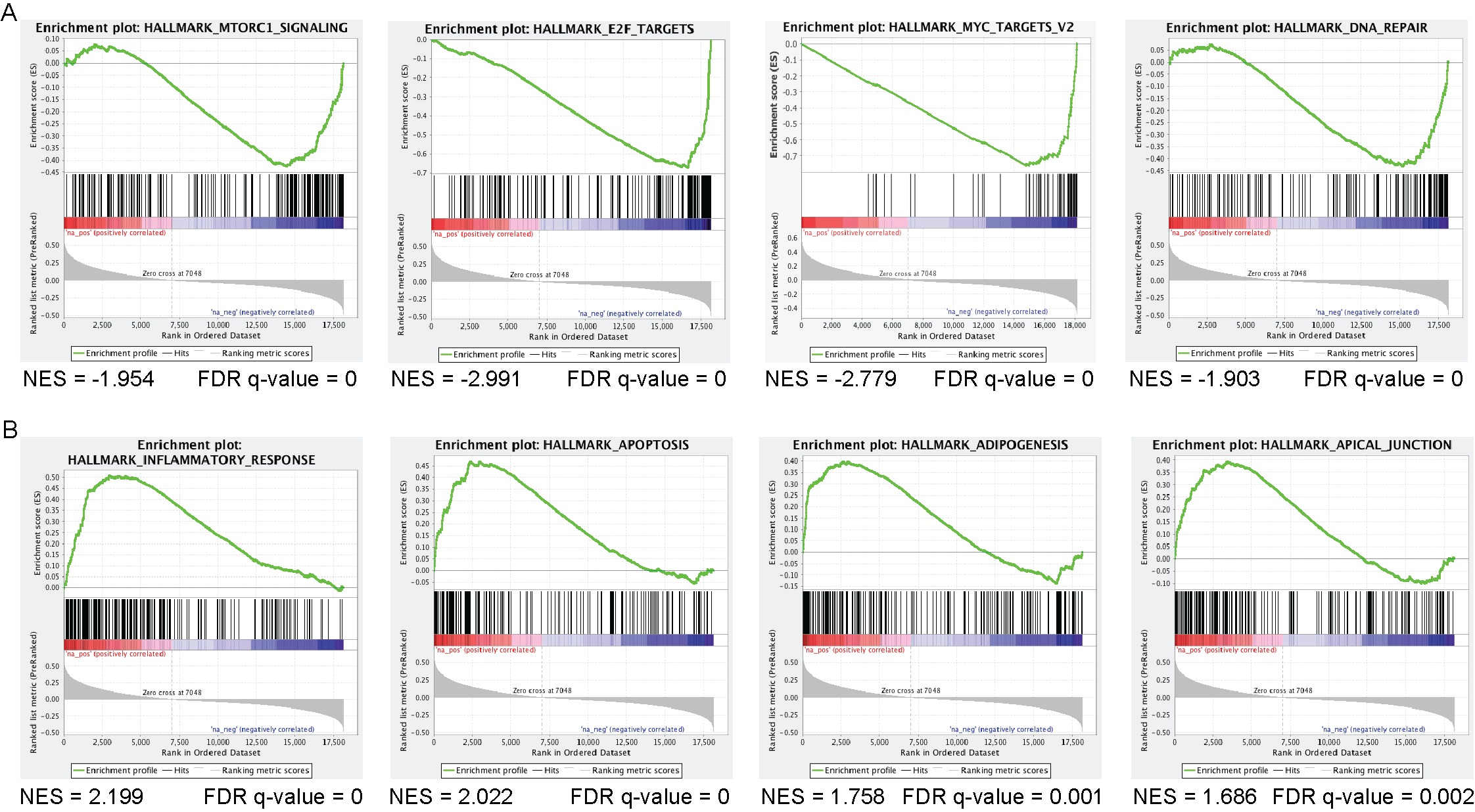

Supplement: S3 Fig — Related to Fig 1. (A) Transcripts positively correlated TXNIP expression across almost 2,000 breast tumors are negatively correlated with genes in the 4 shown Hallmark datasets or (B) positively correlated with genes in the 4 shown Hallmark datasets. (TIF) [file pbio.3001778.s003.tif]

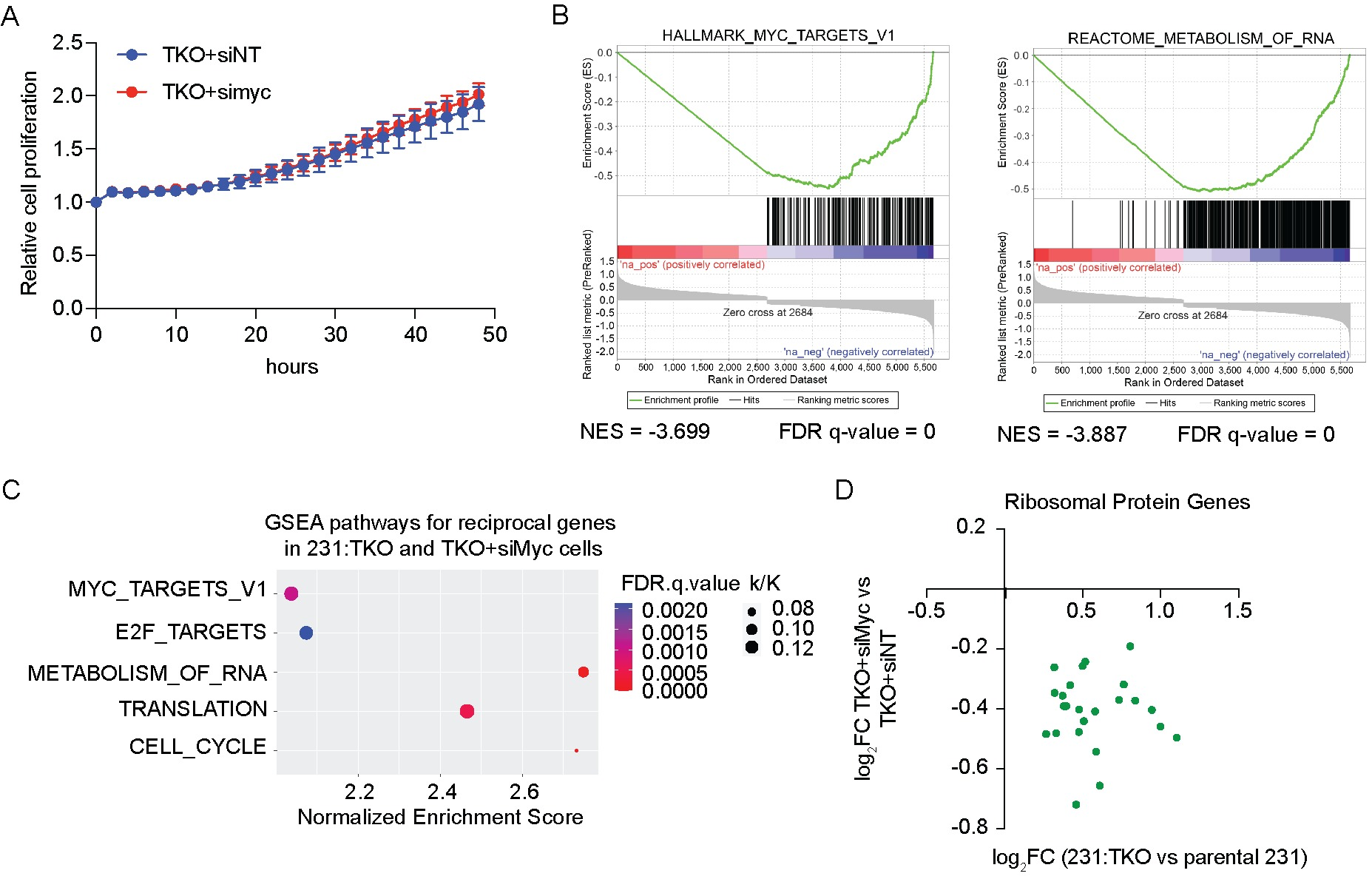

Supplement: S4 Fig — Related to Fig 2. (A) Cell proliferation for 231:TKO+siNT and 231:TKO+siMyc cells in regular medium over a 48-hour time course was measured based on the percentage of confluency using real-time videography. (B) A preranked GSEA was preformed using a ranked list of the differentially expressed genes in 231:TKO+siMyc cells and the indicated Hallmark and Reactome datasets. (C) A ranked list of the 548 reciprocally regulated genes in Fig 2D were used in a GSEA using the MSigDB and the Hallmark and Reactome datasets. k/K value is a ratio of number of genes in our dataset (k) divided by the number of genes in the indicated dataset (K). (D) Expression changes of 24 ribosomal protein genes regulated 231:TKO and 231:TKO+siMyc datasets. The underlying data for S4A, S4C and S4D Fig can be found in S1 Data. GSEA, Gene Set Enrichment Analysis; MSigDB, Molecular Signatures Database; siMyc, siRNA Myc-targeting; siNT, siRNA non-targeting; TKO, TXNIP-knockout. (TIF) [file pbio.3001778.s004.tif]

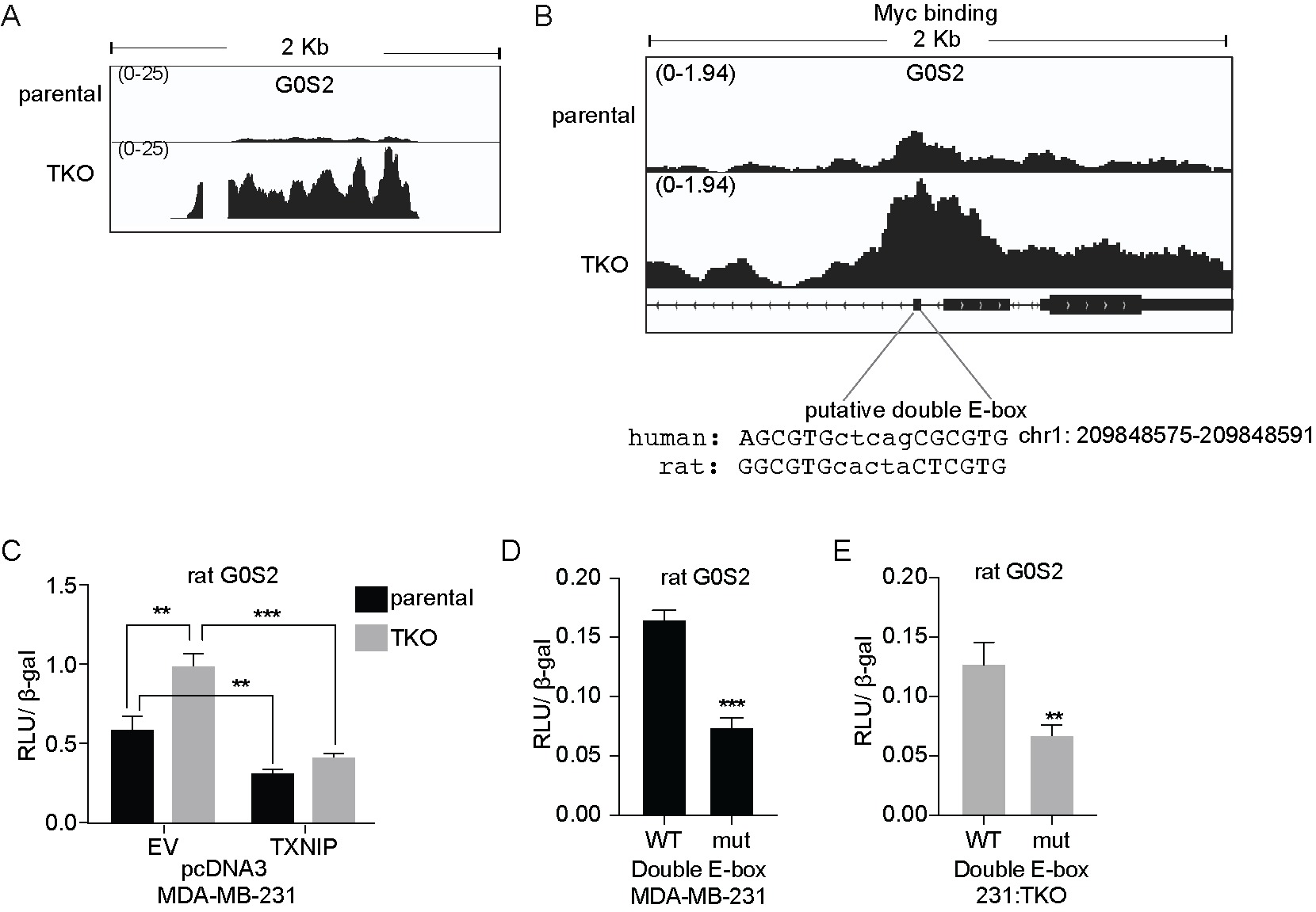

Supplement: S5 Fig — Related to Fig 4. (A) Genome browser view from RNA sequencing of human G0S2 (G0S2) mRNA in parental 231 and 231:TKO cells. (B) Myc binding, as visualized using IGV_2.5.2, to G0S2 in parental 231 and 231:TKO cells. Putative double E-Box element in the G0S2 promoter [41] encompassed the Myc-binding site. (C) Luciferase activities of rat G0S2 reporter in parental and 231:TKO cells with ectopic expression of human TXNIP from pcDNA3 vector or pcDNA3 EV were measured. Luciferase activity was normalized to β-gal activity. At least 2 biological replicates were carried out for all luciferase experiments. Representative figures were shown. Values are reported as mean and standard deviation. **p < 0.01; ***p < 0.001. (D and E) The luciferase activities from WT rat G0S2-luciferase reporter construct and mut rat G0S2-luciferase reporter construct in lysates from parental 231 (D) and 231:TKO (E) were measured. The double E-Box element of G0S2 promoter in the rat G0S2-luciferase construct was deleted using site-directed mutagenesis [41]. At least 2 biological replicates were carried out for all luciferase experiments. Representative figures were shown. Values are reported as mean and standard deviation. **p < 0.01; ***p < 0.001. The underlying data for S5C–S5E Fig can be found in S1 Data. EV, empty vector; mut, mutated; TKO, TXNIP-knockout; TXNIP, thioredoxin interacting protein; WT, wild-type; β-gal, beta-galactosidase. (TIF) [file pbio.3001778.s005.tif]

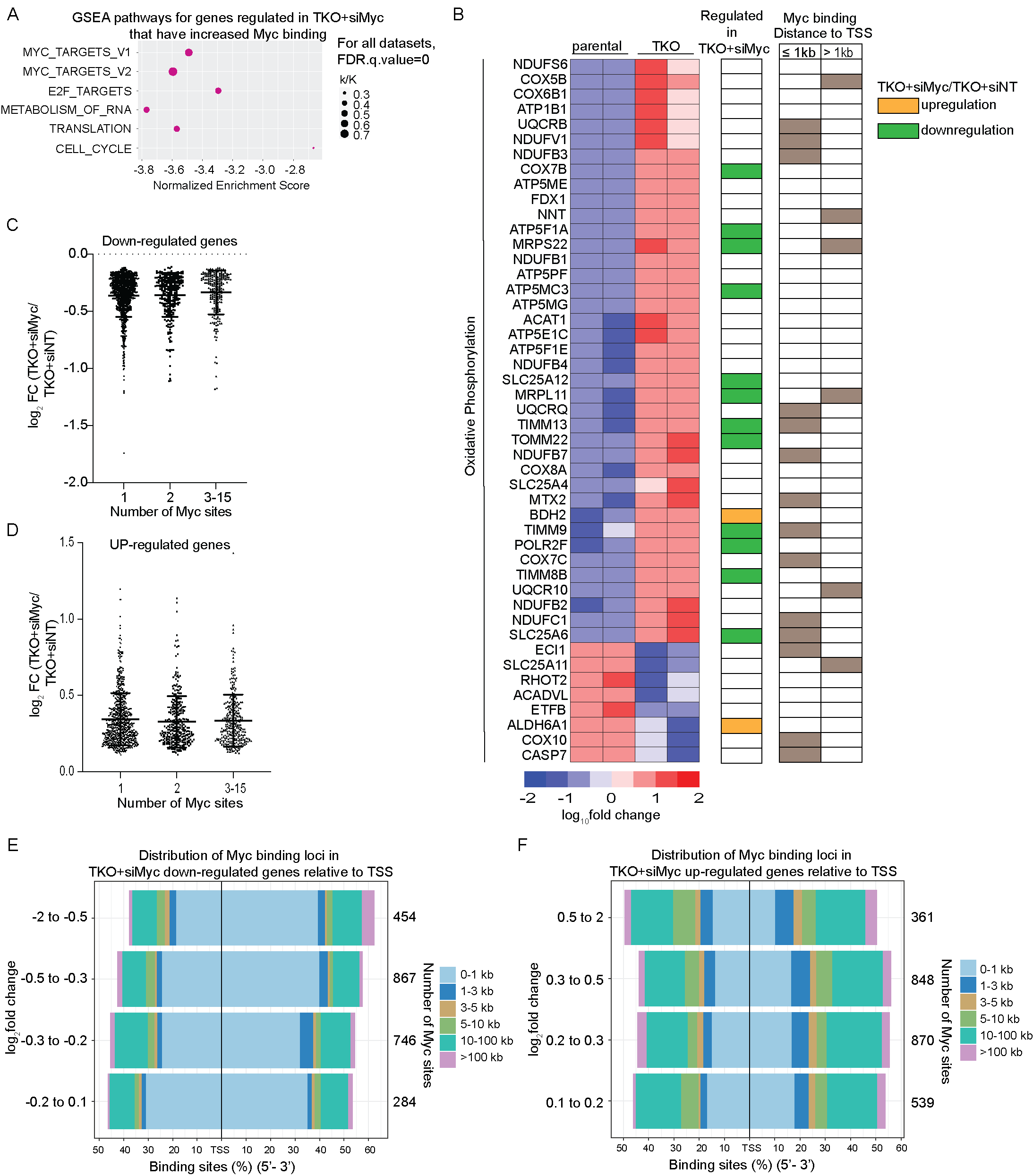

Supplement: S6 Fig — Related to Fig 5. (A) The list of 2,903 genes that showed increased Myc binding in 231:TKO cells compared to parental 231 cells were ranked according to their differential expression in 231:TKO+siMyc cells. This list was analyzed using preranked GSEA to identify enriched pathways in the MSigDB. k/K value is a ratio of number of genes from our dataset (k) divided by the number of genes in the indicated dataset (K). (B) Heatmap of genes regulated in 231:TKO cells that are enriched in the Reactome oxidative phosphorylation dataset. Differential regulation in 231:TKO+siMyc cells are indicated by yellow (up-regulation) or green (down-regulation) boxes. The distances of Myc binding sites from TSS are determined using GREAT [43]. The genes that have a Myc binding event within 1 kb or more than 1 kb from TSS are indicated in brown. Open boxes indicate no Myc binding. (C and D) Differentially down-regulated (C) or up-regulated genes (D) in 231:TKO+siMyc cells were divided into groups based on the number Myc sites associated with each gene. (E and F) The distribution of Myc binding loci relative to the TSS for down-regulated (Myc-activated targets) (E) and up-regulated (Myc-repressed targets) (F) genes in 231:TKO+siMyc cells were annotated using ChIPseeker. The distance to the TSS was then compared change in gene expression following Myc knockdown. The underlying data for S6A, S6C and S6D Fig can be found in S1 Data. GREAT, Genomic Regions Enrichment of Annotations Tool; GSEA, Gene Set Enrichment Analysis; MSigDB, Molecular Signatures Database; siMyc, siRNA Myc-targeting; TKO, TXNIP-knockout; TSS, transcriptional start site; TXNIP, thioredoxin interacting protein. (TIF) [file pbio.3001778.s006.tif]

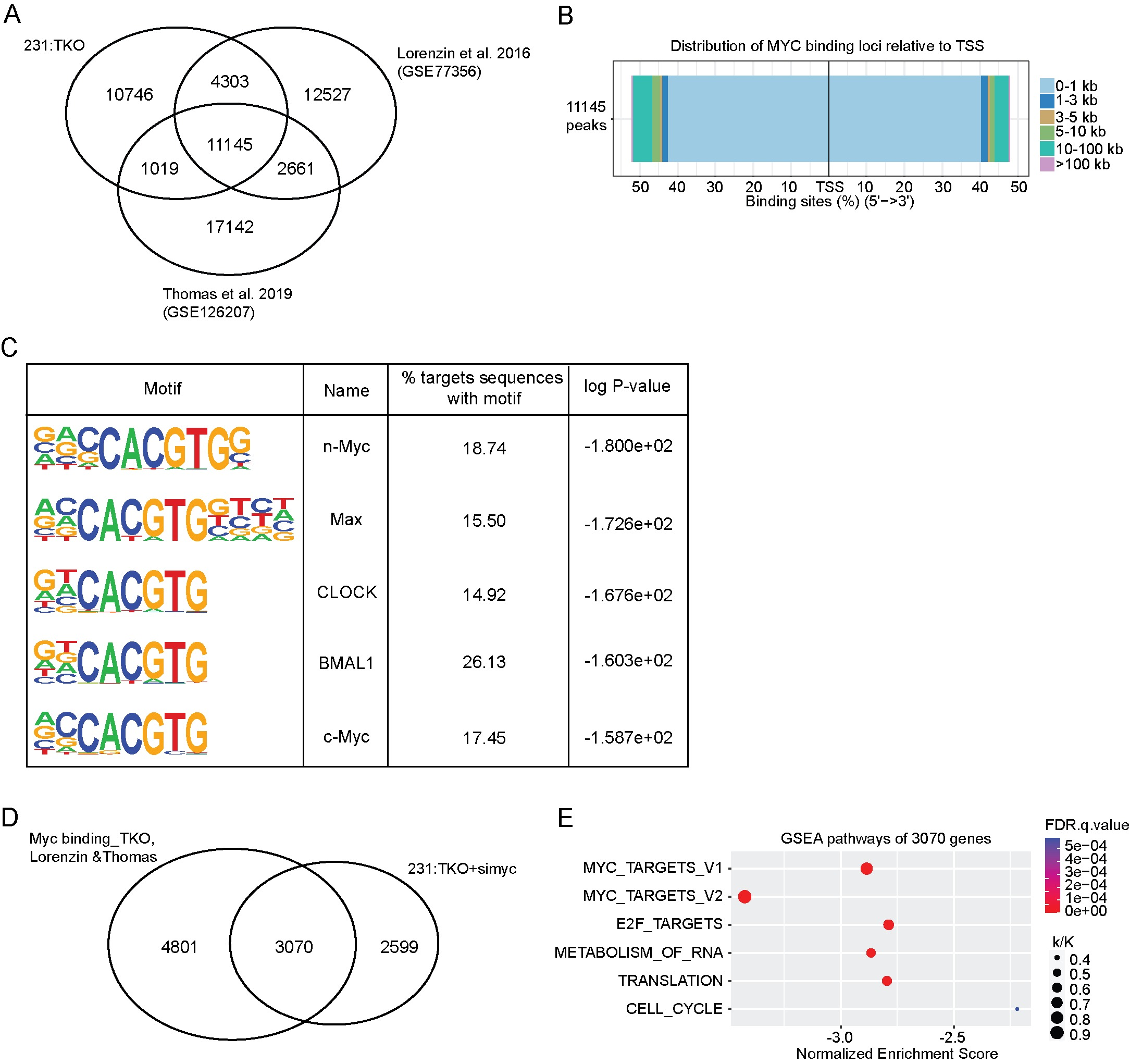

Supplement: S7 Fig — Related to Fig 5. (A) The Myc binding sites in our Myc ChIP-sequencing in 231:TKO dataset was compared with Myc binding sites in published Myc ChIP-sequencing datasets GSE77356 and GSE126207 [27,42]. The narrow peaks of each dataset were compared using bedtools intersect to identify overlapped peaks in the 3 datasets [75]. The Venn diagram was drawn using a VennDiagram package in R studio. (B) The distance of the 11,145 overlapped Myc binding sites from TSS was annotated using the ChIPseeker program. (C) Myc binding motifs were determined using HOMER. (D) A total of 11,145 overlapped narrow Myc-binding peaks were annotated with genes using GREAT [43]. A total of 7,871 genes that are annotated from 11,145 overlapped narrow peaks were compared with regulated genes in 231:TKO+siMyc cells. The Venn diagram was drawn using a VennDiagram package in R studio. (E) Preranked GSEA using a ranked list of 3,070 Myc-dependent and Myc-bound genes and the Hallmark and Reactome datasets in the MSigDB. k/K value is a ratio of number of genes in our dataset (k) divided by the number of genes in the indicated dataset (K). ggplot2 was used to draw dot plots for pathway enrichment. The underlying data for S7E Fig can be found in S1 Data. GREAT, Genomic Regions Enrichment of Annotations Tool; GSEA, Gene Set Enrichment Analysis; HOMER, Hypergeometric Optimization of Motif EnRichment; MSigDB, Molecular Signatures Database; siMyc, siRNA Myc-targeting; TKO, TXNIP-knockout; TSS, transcriptional start site; TXNIP, thioredoxin interacting protein. (TIF) [file pbio.3001778.s007.tif]

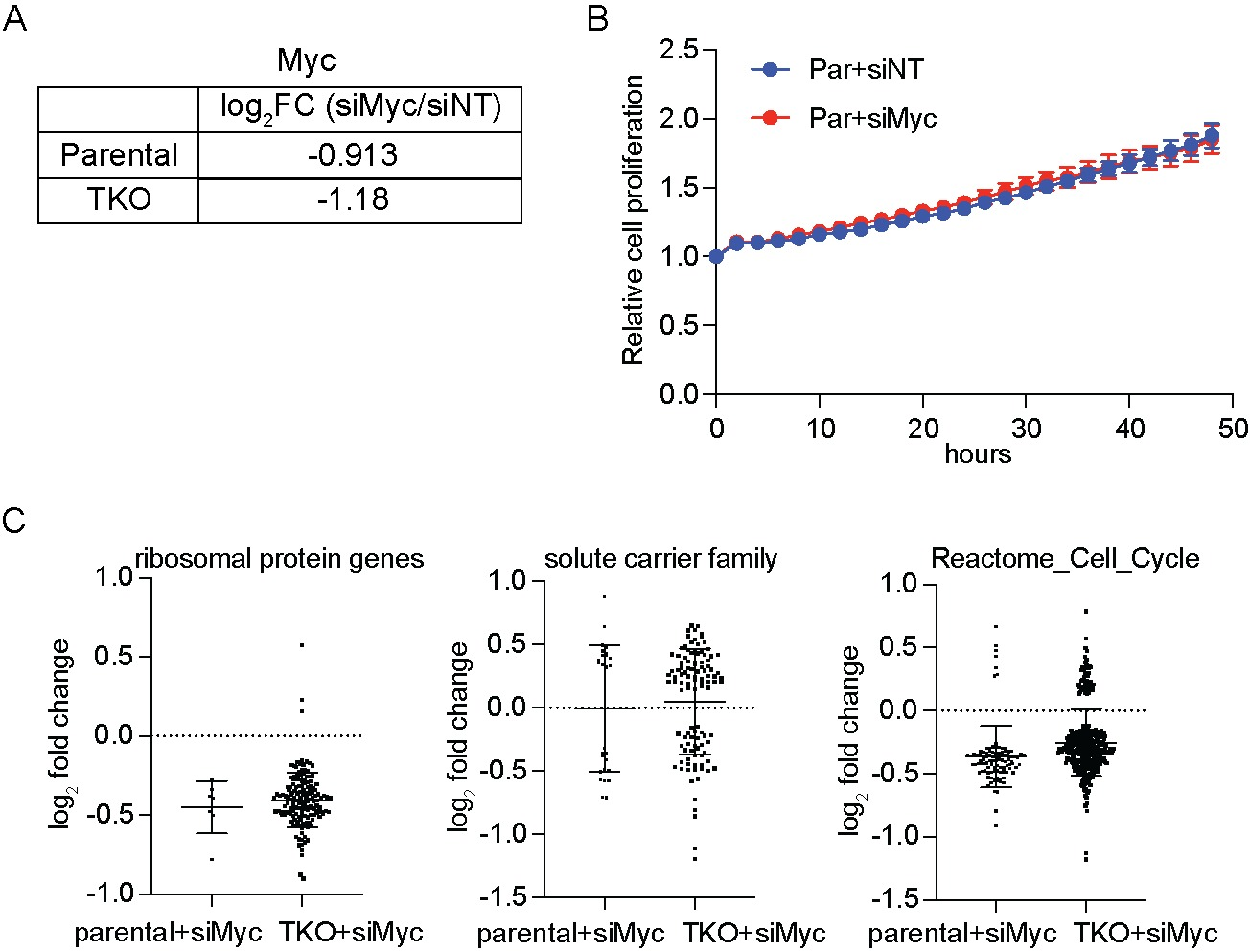

Supplement: S8 Fig — Related to Fig 6. (A) The fold decrease in Myc mRNA by siMyc in both parental 231 and 231:TKO cells was extracted from our RNA sequencing data. (B) Cell proliferation for parental 231 with siNT or siMyc in regular medium over a 48-hour time course was measured based on the percentage of confluency using real-time videography. (C) The differential expression of genes in 3 different functional groups in parental 231+siMyc and 231:TKO+siMyc datasets were compared. The underlying data for S8B and S8C Fig can be found in S1 Data. siMyc, siRNA Myc-targeting; siNT, siRNA non-targeting; TKO, TXNIP-knockout; TXNIP, thioredoxin interacting protein. (TIF) [file pbio.3001778.s008.tif]

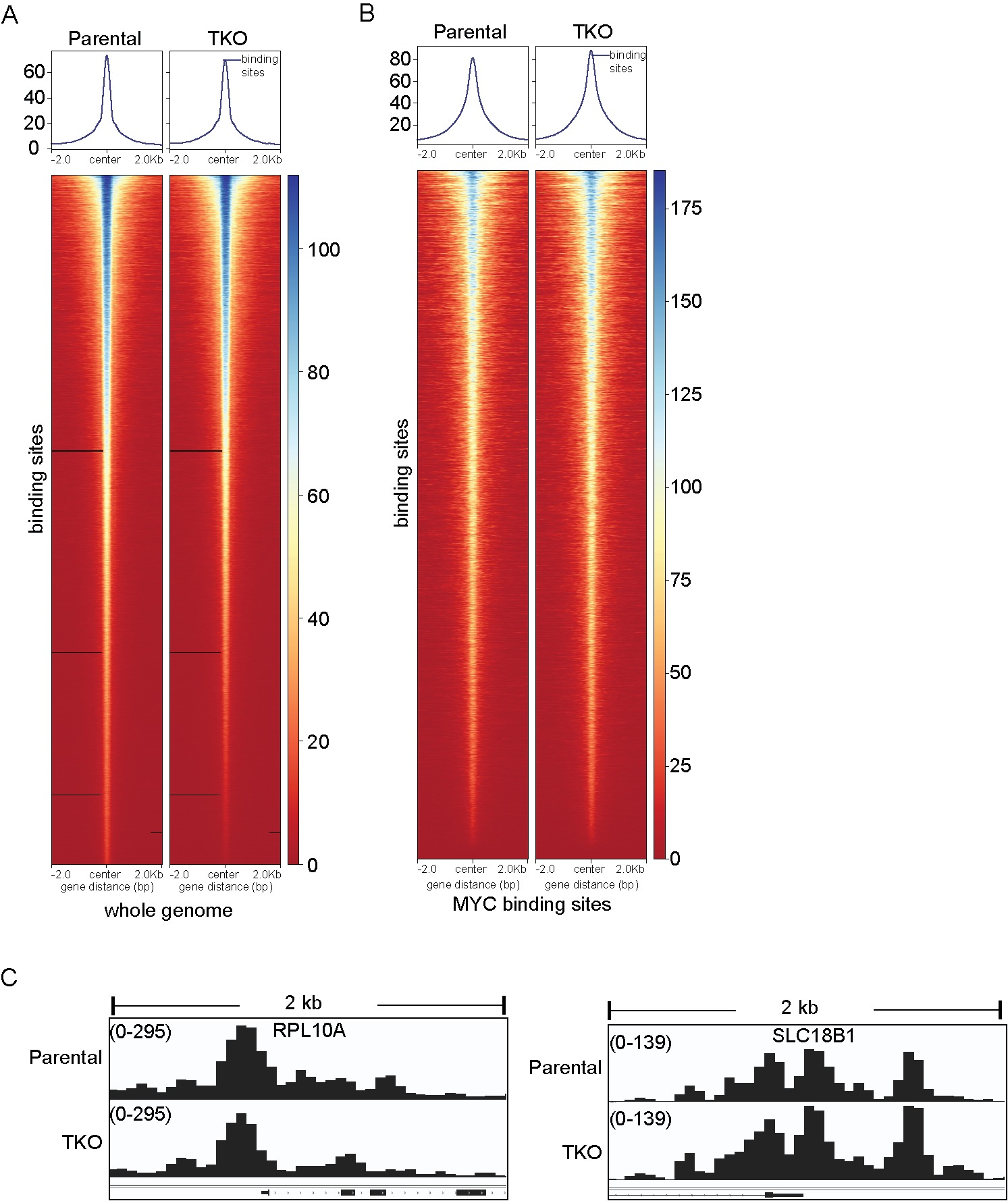

Supplement: S9 Fig — (A and B) To determine the accessibility of chromatin, we used to perform ATAC-seq in both parental 231 and 231:TKO cells. Heatmaps showing the chromatin accessibility in parental 231 and 231:TKO across the entire genome (A) and at Myc-binding sites (B). Heatmaps were generated within 2 kb upstream and downstream of the accessibility peaks using a p-value cutoff of 0.01. (C) Genome browser views of chromatin accessibility in the proximity of RPL10A and SLC18B1 in parental 231 and 231:TKO cells. ATAC-seq, assay for transposase-accessible chromatin using sequencing; RPL10A, ribosomal protein L10a; SLC18B1, solute carrier family 18 member B1; TKO, TXNIP-knockout; TXNIP, thioredoxin interacting protein. (TIF) [file pbio.3001778.s009.tif]

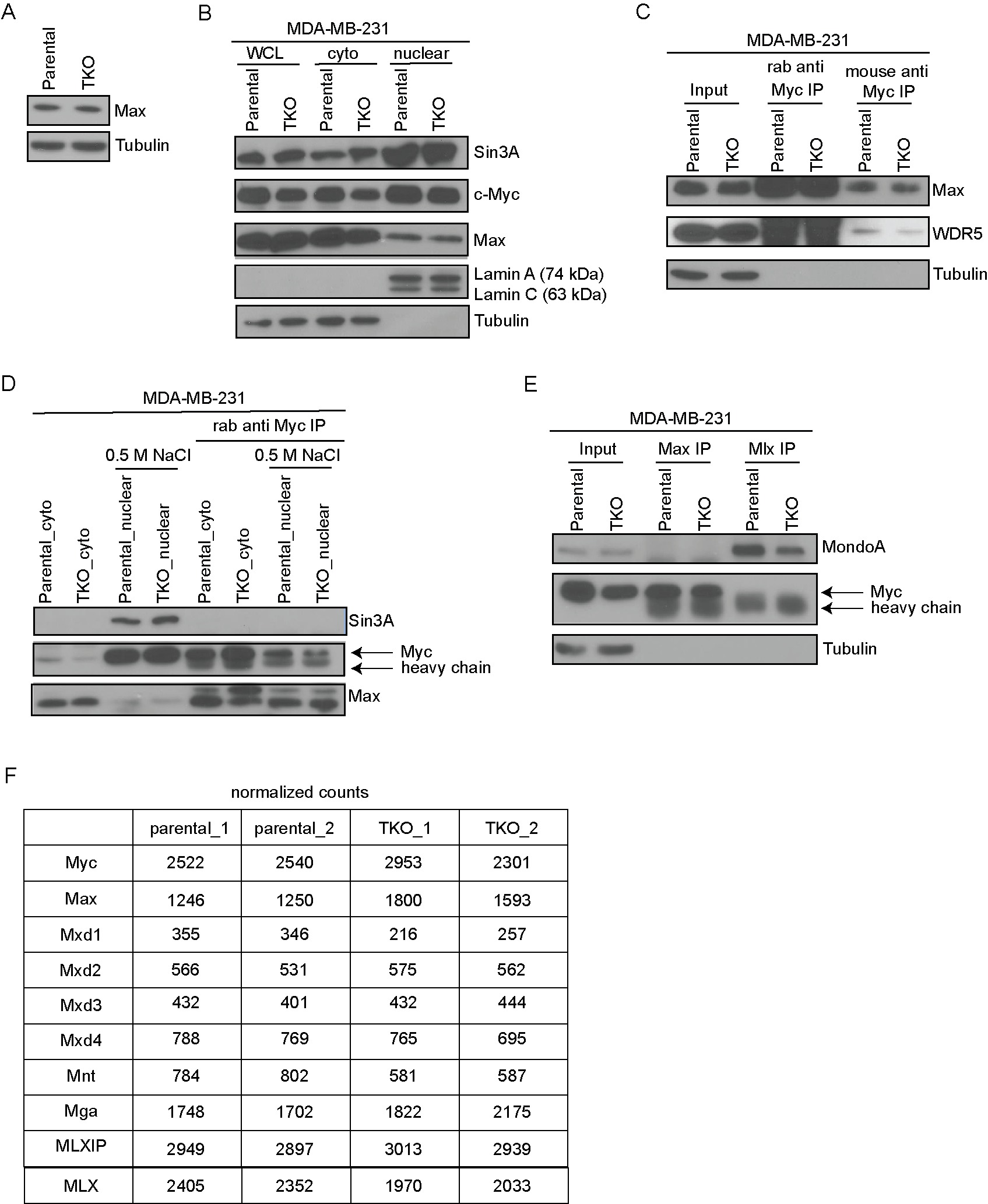

Supplement: S10 Fig — (A and B) Western blotting was used to determine the levels of indicated proteins in whole cell lysates (A), cytoplasmic and nuclear fractions (B) from parental 231 and 231:TKO cells. (C) Myc protein was immunoprecipitated with c-Myc antibodies from rabbit (rab) and mouse, respectively. Associated proteins were detected by immunoblotting. (D) Max protein was co-immunoprecipitated with c-Myc antibodies from the cytoplasmic (cyto) and nuclear fractions of parental 231 and 231:TKO cells. The levels of immunoprecipitated Max in these cell lines were assessed by western blotting. (E) Max protein was immunoprecipitated with Max antibody. Associated proteins were detected by immunoblotting. A Mlx IP in was used as a specificity control. The indicated immunoprecipitated proteins were examined by western blotting. (F) Expression levels of the members of the extended Myc family of transcription factors expressed as normalized counts from our RNA-seq analysis. IP, immunoprecipitation; TKO, TXNIP-knockout; TXNIP, thioredoxin interacting protein. (TIF) [file pbio.3001778.s010.tif]

Fig 1A

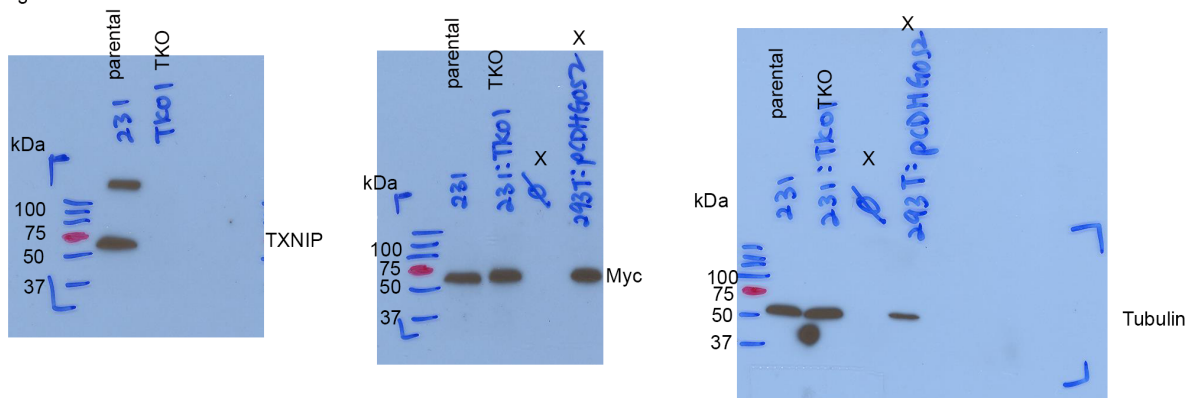

Fig 2A

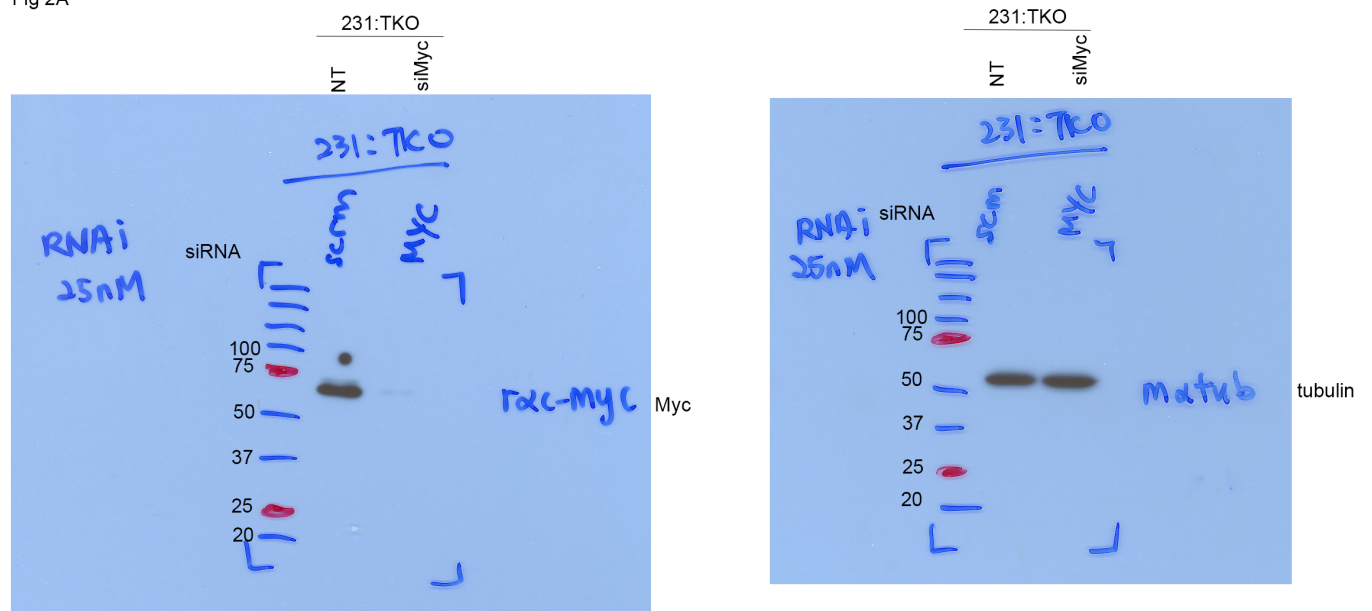

Fig. 4B

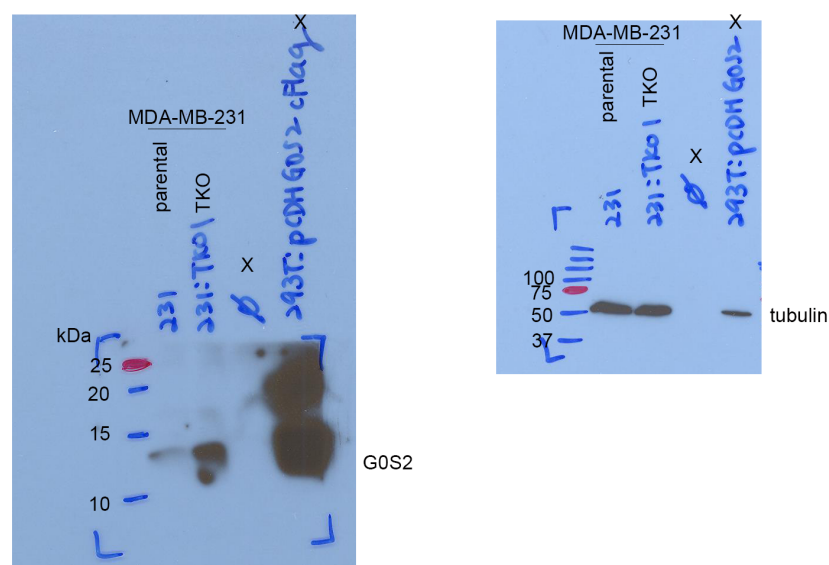

Figure 4E

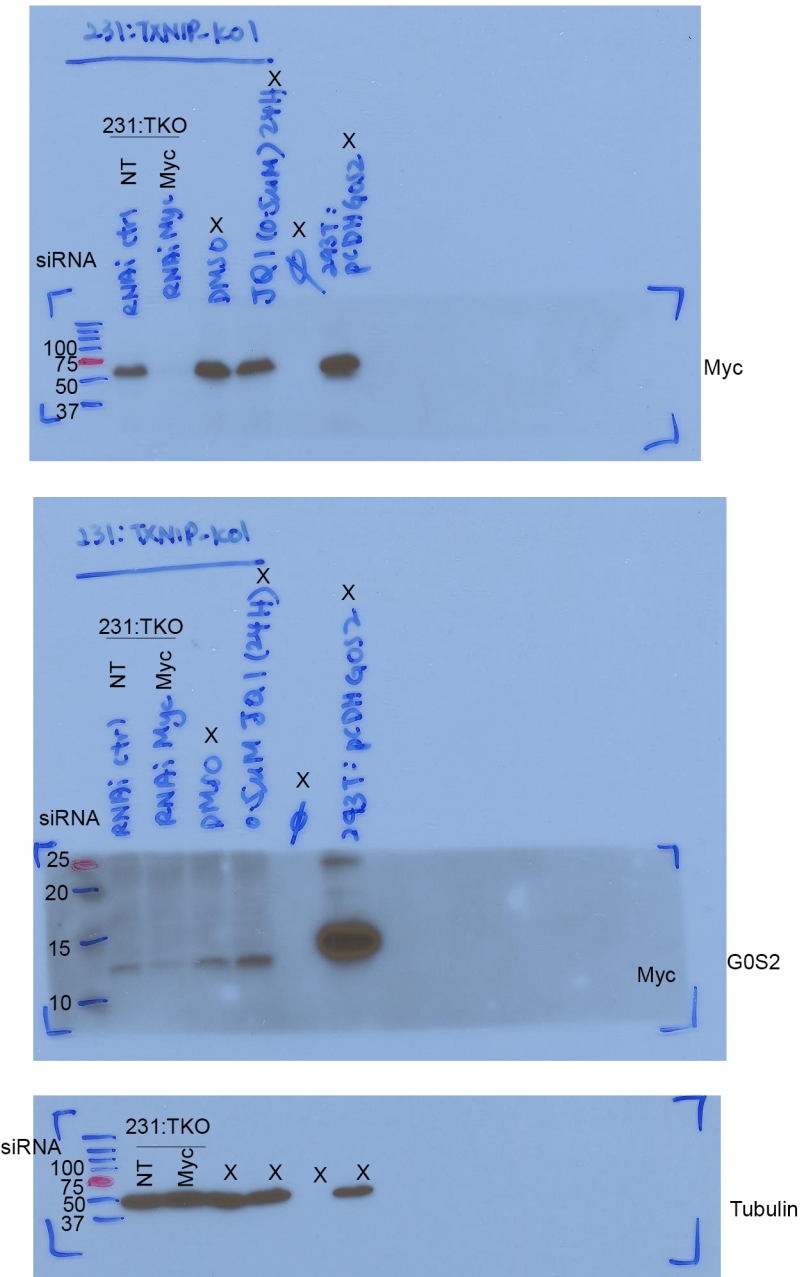

Figure 6A

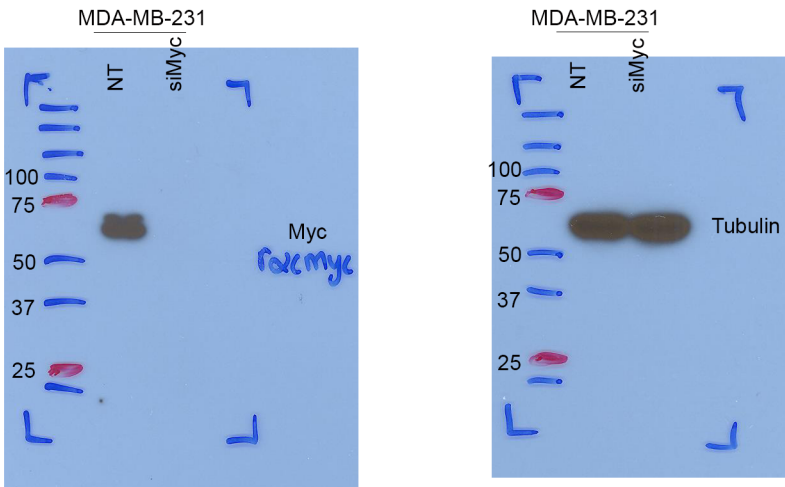

S1C Fig.

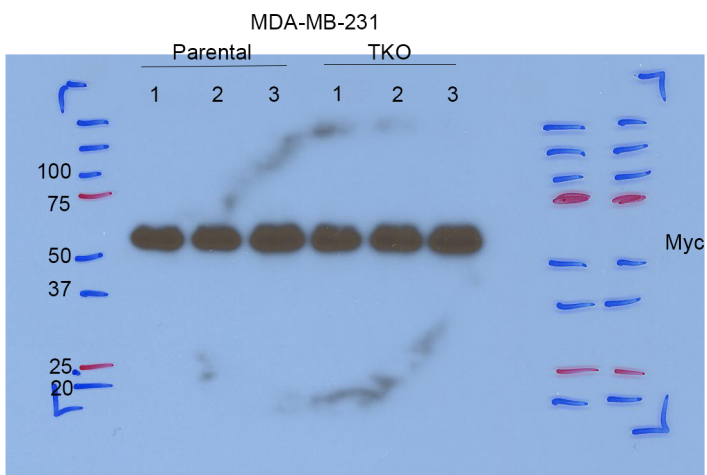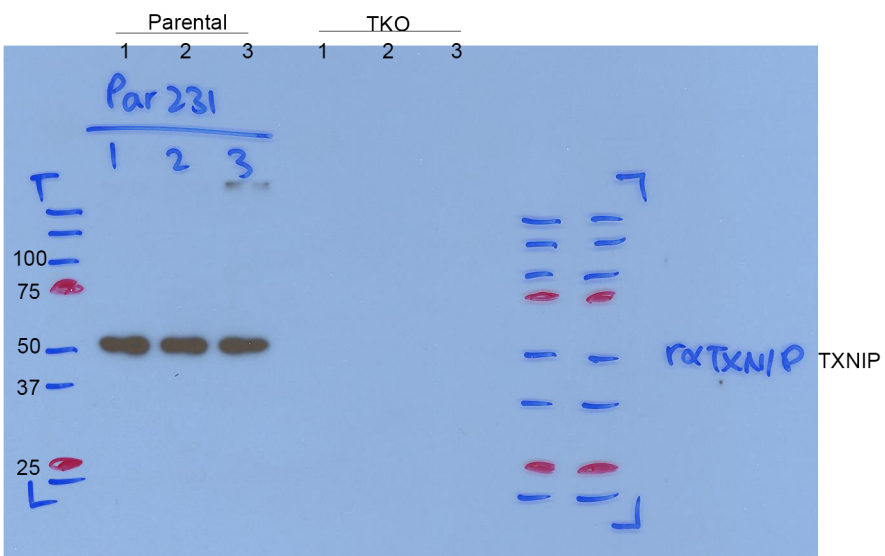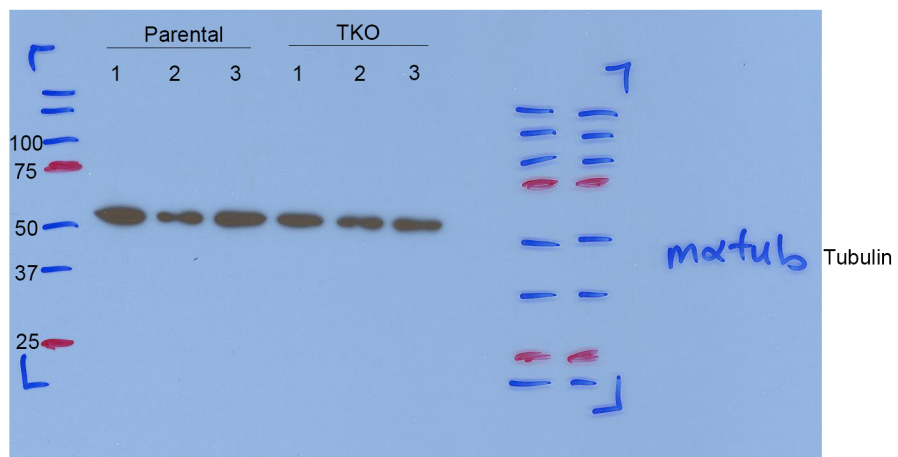

S2A Fig.

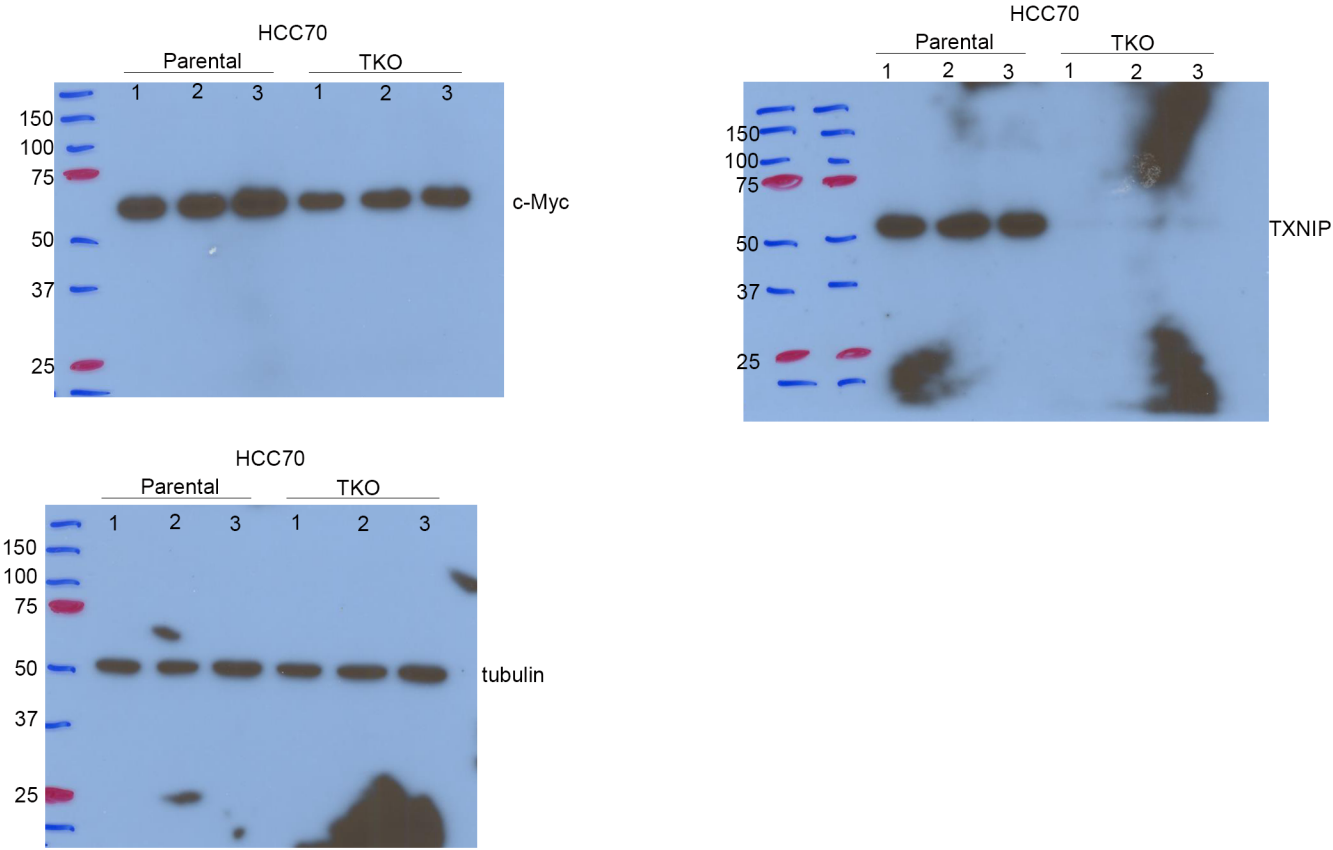

S2D Fig.

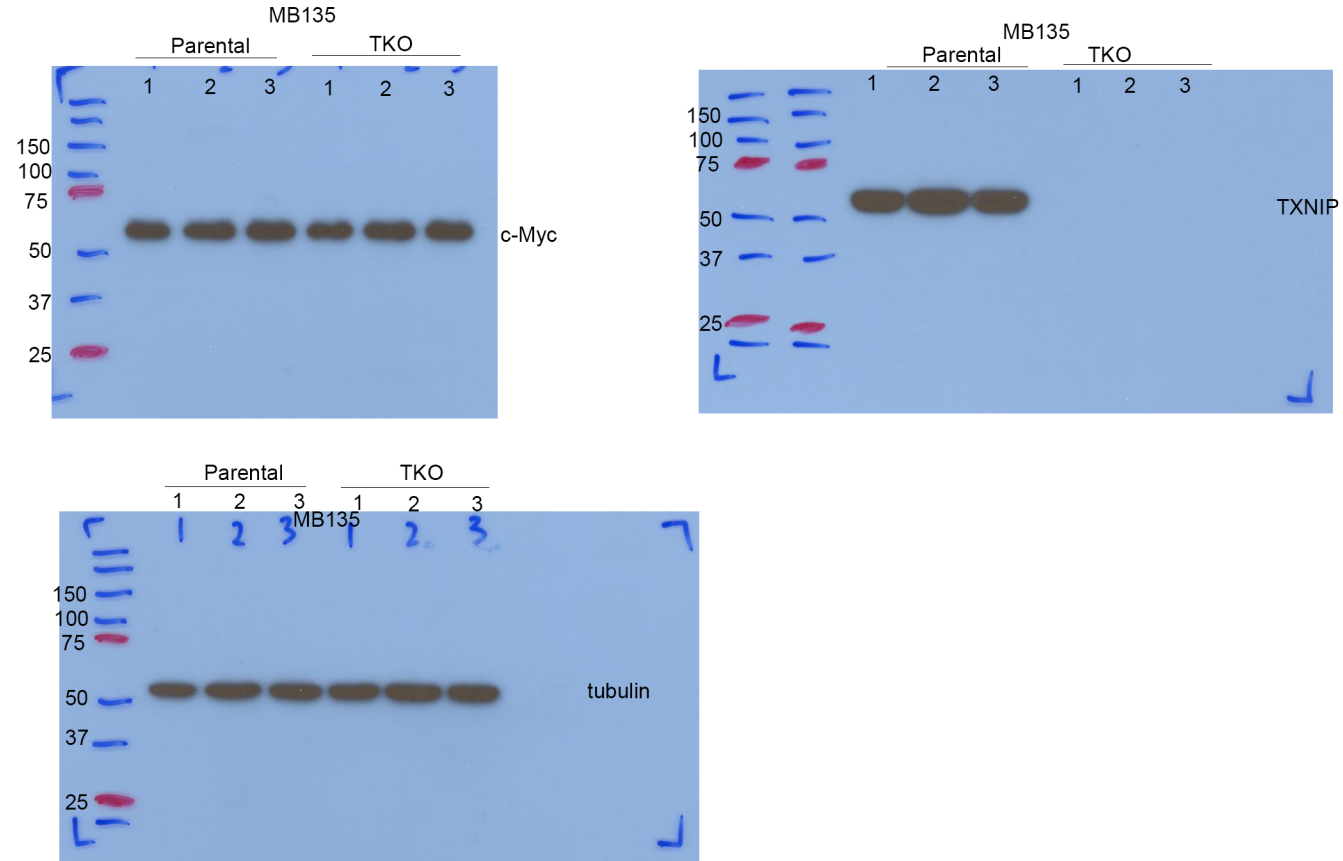

S10A Fig.

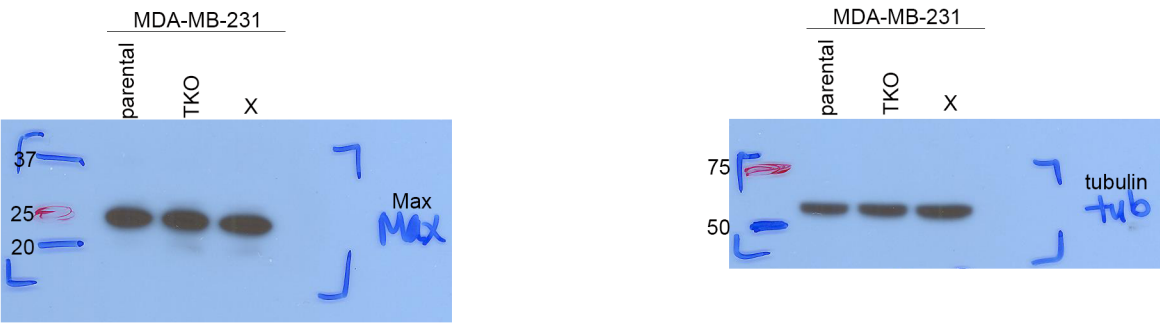

S10B Fig.

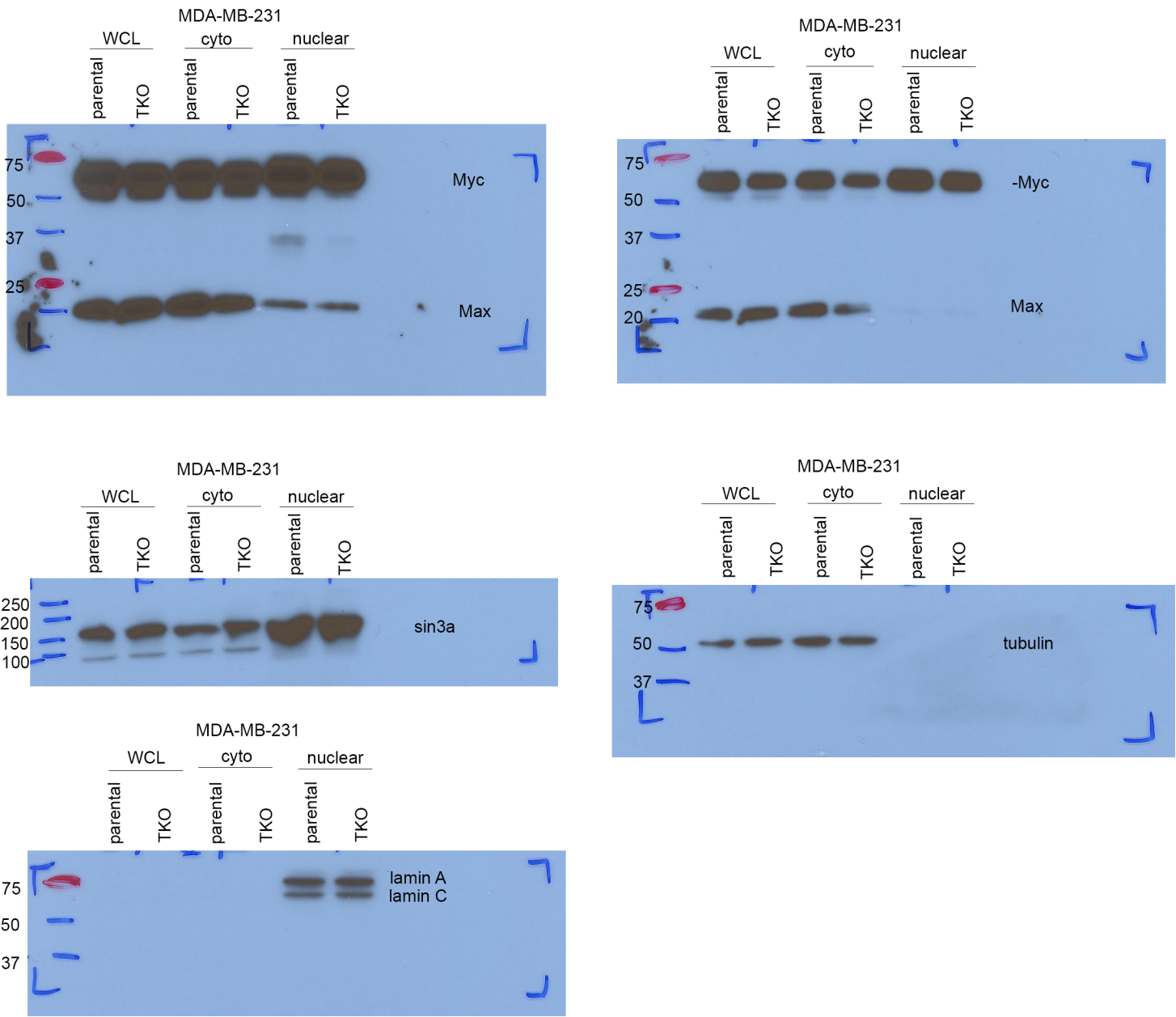

S10C Fig.

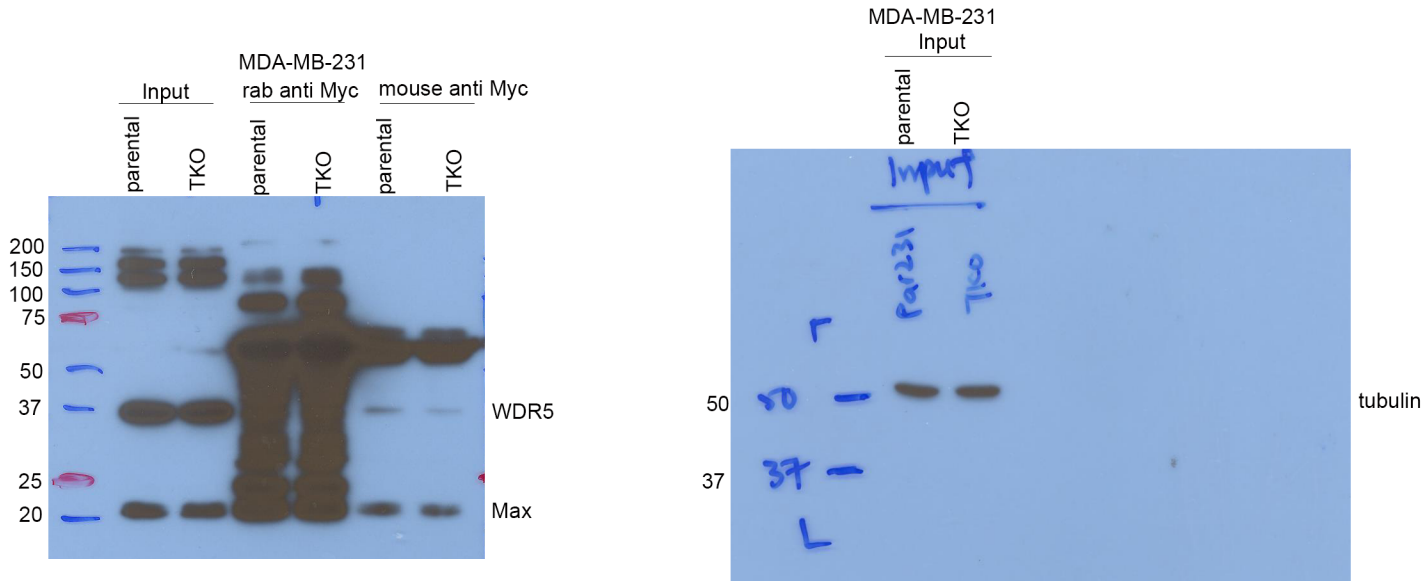

S10D Fig.

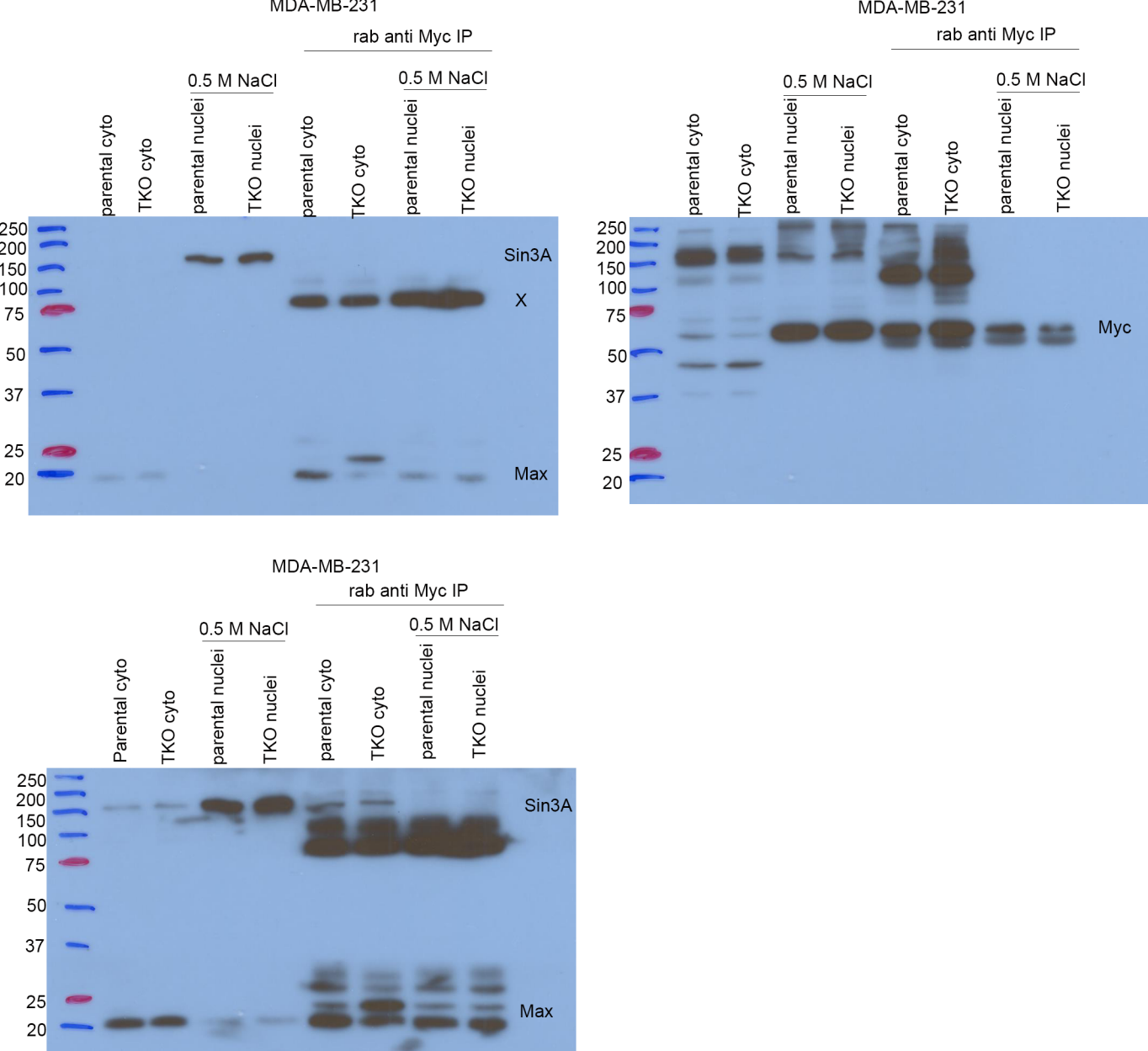

S9F Fig.

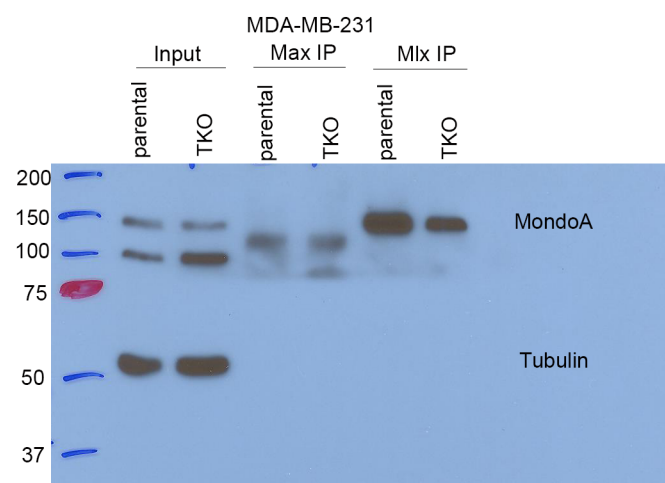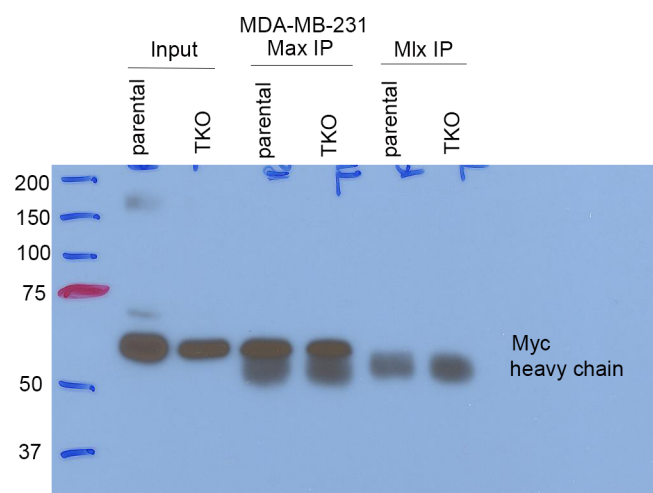

Supplement: S1 Raw Images — (PDF) [file pbio.3001778.s012.pdf]
