# Supplementary material for: TXNIP loss expands Myc-dependent transcriptional programs by increasing Myc genomic binding
Source: PLoS Biol. 2023 Mar 17;21(3):e3001778. doi: 10.1371/journal.pbio.3001778 (PMC10058090; doi:10.1371/journal.pbio.3001778)
Supplement: Attachment — Submitted filename: PBIOLOGY-D-22-01559R2_RESPONSE_1-20-2023.docx [file pbio.3001778.s013.docx]

Response to Reviews for PBIOLOGY-D-22-01559R1

Dear Richard, we would like the resubmission of our manuscript “TXNIP loss expands Myc-dependent transcriptional programs by increasing Myc genomic binding" (PBIOLOGY-D-22-01559R1) to be reconsidered for publication in PLoS Biology. We received the initial evaluation of our manuscript on September 9^th^, 2022 and, while declined at that time, you invited a resubmission within 6 months. Below we provide a point-by-point responses to each the reviewer’s critiques of our manuscript. In response to reviewer 3’s open-ended comments, we provide RNA-seq experiments from one additional TXNIP KO cell line, new metabolomics data and clarification of all of his points. We have performed several xenograft experiments showing that TXNIP is required for orthotopic growth of MDA-MB-231 cells in immune-compromised mice. There are many interesting, but still open questions, regarding these experiments. Given this, and the current length of the manuscript, we have elected to not present this xenograft data in the paper. We have provided this data in this response letter, so it can be evaluated by reviewer 3 and the other two reviewers. We are grateful that the reviewers found our results to be novel and interesting. Their comments have strengthened the manuscript considerably. As you will see, we have added several new experiments (RNA-seq from an additional TNBC cell line, metabolomics analysis of control and TXNIP knockout cells, analysis of other published ChIP seq experiments, all of the requested proliferation experiments, all of the requested western blots and their quantification, an ATAC-seq experiment and multiple experiments examining levels of Myc, Max and Myc:Max complexes) and have made a considerable effort to address each of the reviewers’ concerns. We hope that our manuscript is now acceptable for publication in PLoS Biology.

We appreciate that the precise molecular details of how TXNIP loss increases Myc genomic activity remain to be described. We have tested many of the obvious hypotheses that might provide us with a deeper mechanistic understanding. At the request of reviewers 1 and 2, we have provided this negative data in two supplemental figures. Rest assured that deciphering how TXNIP controls global Myc genomic binding is front and center in our minds, but we believe such a mechanistic dissection is beyond the scope of this current work.

Sincerely, Don Ayer

**Reviewer #1:**

**Major points1**

*1. The gene sets described in Fig. 1B other than "Myc Target Genes" represent many functions that nonetheless have been described as being Myc-regulated (i.e. mitochondrial structure/function, translation, cell cycle). How many of the genes within these sets are known to be direct Myc targets?*

Our RNA-seq data revealed 1050 transcripts upregulated and 742 transcripts downregulated in 231:TKO cells relative to parental 231 cells. About 50% of these genes have an associated Myc binding site, suggesting that their regulation is direct. Similarly, our Myc-knockdown experiment in 231:TKO cells identified 5669 Myc-dependent genes (Fig 2C), of which, 2093 are associated with a Myc binding site (Fig 5A). These 2093 genes potentially identify the direct Myc-dependent transcriptome in 231:TKO cells and are enriched in known Myc targets, E2F targets, and pathways involved in translation, metabolism of RNA and cell cycle (S6A Fig). Thus, Myc appears to directly regulate roughly 2000 genes in 231:TKO cells that are associated with progrowth pathways that are well established to be Myc-regulated. At this level of analysis and given the variance in gene expression programs between tumor type and/or cell lineage, we believe that understanding Myc-involved pathways is more informative than determining the particular Myc targets in TKO:231 cells have been shown to be Myc-dependent in other cell models. Further there a limited number of studies that have combined conditional Myc expression with a ChIP-seq analysis, and none of these has been performed in TNBC cells, to identify potential direct Myc targets.

At the advice of the reviewer, we evaluated the Myc ChIP-seq experiments in ENCODE data. All of these datasets were flagged by the consortium for having a number of technical issues, e.g., low read counts and/or low complexity libraries. However, to see how our Myc genomic binding profile in 231:TKO cells compared to others in the literature, we downloaded and analyzed Myc ChIP-seq datasets from U2OS and Ramos cells. Using narrow peaks files and the intersect bed tool, we discovered that about 30% of the Myc binding sites (11,145 sites) in each dataset were conserved between all three experiments. These 11,145 sites are associated with 7871 genes. As one might expect these shared binding sites are highly enriched for consensus Myc-binding sites and more than 80% are located within 1kb of the TSS of the associated gene . Further, 3070 (~40%) of these 7871 genes were regulated in a Myc-dependent manner in 231:TKO cells and these genes are enriched for Myc-dependent targets and progrowth pathways similar to our other experiments. We describe this comparative analysis in the results (lines 447-459) and present the data in a new supplemental figure (S7 Fig). Collectively, these findings show that our new data in 231:TKO cells is consistent with data generated in other cell types.

*2. The authors should more thoroughly discuss the fact that the numbers of both positive and negative Myc targets seem to increase in response to TXNIP inactivation.*

We have expanded the discussion to address this point (lines 722-730). Our data shows that Myc binding events occur in about 50% of both up- and down-regulated genes in the 231:TKO cells. This finding suggests that at least some of the “negative” Myc targets, may be repressed by a direct Myc-dependent mechanism, while the others may be regulated by an indirect mechanism. In terms of a direct mechanism(s), we do not see enrichment of Miz1 binding sites in the proximity of Myc-binding peaks and SP1 sites are only found in proximity of about 10% of Myc-binding peaks. This finding implies other Myc-dependent mechanisms (at least at Myc bound genes) must be responsible for most of the Myc-dependent downregulated transcripts we observed in 231:TKO cells.

*3. Could the increase in Myc-dependent transcription we observe be attributable to changes in Myc:Max complexes.*

Reviewer 2 had the same comment. We do not see obvious changes in the level of Myc:Max complexes. We have included several panels of new supplementary data in the discussion to address this important point (S9 and S10 Fig). We note that there are not good antibodies against many of the members of the extended Myc family so we have not assessed their protein levels directly. We have also expanded the discussion (lines 674-680) to include this data and a discussion of the potential for heterocomplex “shuffling” with other members of the extended Myc network.

*4. The reviewer wondered if redox status was altered by TXNIP loss and whether this might account for the increase in Myc binding we observe.*

We used 2,7-dichlorodihydrofluoroscein diacetate (DCFDA) to test ROS levels in parental 231 and 231:TKO cells. We identified an ~25% decrease in ROS that might be attributable to TXNIP loss in 231:TKO cells. This modest reduction in ROS might contribute to the increase in Myc genomic binding we observe, but this finding merits further study which we believe is beyond the scope of this manuscript. We elected not to include this data in a supplement as it doesn’t provide clarity. We do provide this data here for the reviewers’ consideration.

**Minor Points**

*Fig. 1E shows a FDR q value of <0 which doesn't make sense. The actual p value should be indicated. In addition, for C and D, both P values are >0.05.  The authors should state in the figure legend the criteria used that allow them to justify representing these q values as indicating significant enrichment*.

We have corrected the error on Figure 1E (now Figure 1F). We have removed the original Fig 1D to add new data from HCC70 cells. For Figure 1C (now 1D), the FDR q-value is 0.0741. The FDR q-value is adjusted for multiple hypothesis testing and gene set size. Given the relatively small number of gene sets being analyzed, particularly with the HALLMARK database, a more “relaxed” FDR q-value is appropriate. The original Gene Set Enrichment Analysis paper suggests that FDR q-values of <0.25 are appropriate and suitable for hypothesis development.

*Fig. 3. Are the 3 clusters distinguished in anything other than the distance their Myc binding sites are from the TSS of their respective genes and the affinity of these sites for Myc? From these data, is it fair to say that, as a rule of thumb, low-affinity Myc binding sites tend to be located progressively further away from the TSS?*

Our data doesn’t allow us to make this generalization. The data in Figure 3D shows that about 70% of the Myc binding sites in the high affinity cluster 1 are located within 1kb of the TSS of the associated gene. The percentage of Myc binding sites in low affinity cluster 3 are located within 1kb of the TSS of the associated gene drops to 45%. Since the number of Myc binding sites in cluster 3 is greater than the number of Myc binding sites in cluster 1, there are actually more low affinity Myc sites within 1kb of the TSS than high affinity sites. It is absolutely true that there are more low affinity Myc sites are located 10-100kb from the TSS of the associated gene than high affinity sites; however, given the density of low affinity sites in the proximity of the TSS, we don’t believe this observation represents a general trend.

*Fig. 3F & line 270. The authors should explain to the more general reader what the k/K ratio represents.*

We have defined the k/K ratio in each figure legend with a dot plot that contains this type of data.

*Line 330-332. The authors refer to a ChORE element in the rat GOS2 gene that is just upstream of the TSS and that is presumably the functional equivalent of the somewhat degenerative element they identified in the human GOS2 gene's promoter (lines 307-308). How related/conserved are these two elements?*

The double E-box element is conserved between the rat and human G0S2 promoter. We have included an alignment of this double E-box in S5B Fig.

*Line 377: The title of this section is: "TXNIP Controls Myc Transcriptional Programs by increasing Myc Binding". This should be changed given that it is the LOSS of TXNIP that increases Myc binding (lines 396-397: "These results suggest that TXNIP loss increases Myc-dependent gene expression by increasing Myc genome occupancy”.*

We have made the requested change in the text.

**Reviewer #2:**

**Major (essential) points:**
*1. The comparison between 231:TKO siMYC and 231 parental siMYC is complicated by different knockdown efficiencies of MYC.*

We provide western blot data in Figures 2A and 6A showing that siRNA mediated Myc-knockdown in the parental 231 and 231:TKO cells reduces Myc levels to below the level of detection. Further, analysis the RNA-seq data from the two Myc knockdown experiments, indicated that Myc mRNA levels were reduced ~ 2-fold in both parental 231 and 231:TKO cells (S8A Fig). Together these data suggest that Myc knockdown in both cell lines is comparable and a direct comparison between the two cell lines is appropriate.

*2. Authors should support their findings in Figure 6 with an additional method of MYC inhibition, such as expression of OmoMyc or use of a MYC-targeting pharmacologic agent such as MYCMI.*

We have performed the requested experiment using the Myc-targeting agent MYCMI-6. As with the Myc knockdown, MYCMI-6 reduced the transcript levels of the three test genes only in the 231:TKO cells and not the parental 231 cells. This data is shown in Figure 6G.

*3. The mechanism of how TXNIP regulates MYC remains unclear and the reviewer requested the negative results in the supplement*.

We have included several panels of data in new Supplemental Figures 9 and 10. In Supplementary Figure 9, we provide an ATAC-seq experiment that shows that TXNIP loss does not increase chromatin accessibility globally or at Myc targets. As pointed out by the reviewer, this result does not support the model that Myc is a global amplifier of transcription. We have included a new paragraph in the discussion (lines 711-722) to highlight the two models of Myc-dependent gene expression and that our data is most consistent with TXNIP loss increasing Myc’s activity as a sequence specific transcription factor.

*4.* *I believe that Figure 6C is the most important panel in the entire paper, as it shows that the degree of expression regulation by MYC is not changed in the 231:TKO siMYC versus 231 parental siMYC despite the different numbers of MYC targets. However, this panel is somewhat difficult to read and interpret. In addition to 6C itself, it would be useful for the Authors to show these data another way, such as overlapping histograms showing degree of expression in the two models.*

Our Myc knockdown experiment identified 1045 genes that are Myc-dependent in both parental 231 and 231:TKO cells (Fig 6B). To make this clearer, we have included a new panel that shows that the magnitude of Myc-dependence of this group of genes is nearly identical in both cell lines (Fig 6D). We then provide a XY plot (Fig 6E), that shows the fold change and the direction of change following Myc knockdown in each cell line is nearly identical at the single gene level. We have modified the text and the figure to clarify these points.

*5. For the same reason that it was important to include a proliferation assay for 231:TKO cells (Figure S1D), the Authors must include proliferation assays for other manipulations, such as the 231:TKO + siMYC cells and the parental 231 siMYC cells.*

We have conducted cell proliferation assays on the parental 231 and the 231:TKO cells +/- siMyc knockdown. We have also included this data for the HCC70 and HCC70 TXNIP knockout cells we generated and analyzed for this revision. We have not knocked Myc down in the MB135 cells, but have also determined the effect of TXNIP loss on their proliferation. These experiments show that TXNIP knockout doesn't affect proliferation of MDA-MB-231, HCC70 or MB135 cells (S1D, S2C and S2F Fig). Myc knockdown in parental 231 (S8B Fig) or 231:TKO (S4A Fig) cells doesn't affect proliferation during the 48-hour time course of the knockdown. Together, these findings suggest that changes we see in Myc-dependent gene expression result from changes in Myc activity rather than by a secondary mechanism involving cell cycle alterations that stem from reductions in Myc.

**Requested changes to the methods**

*1) How TXNIP mRNA was used to rank the levels of all other genes in 1904 breast cancer lines, and to create a single pre-ranked list for GSEA analysis.*

We have expanded the text in the results (lines 128-135) and added additional details to the methods section (lines 825-830) to clarify our approach.

*2) How GSEA results throughout the paper were identified for presentation. Were these based on rankings of NES strength, or hand-curated from a larger list of results?*

To maintain consistency throughout the paper we hand-curated the enriched pathways from the GSEA pathway analysis. We have clarified this in the text (lines 212-214)

**Minor (non-essential) points:**

*The Authors should discuss why MDA-MB-231 was chosen, particularly with regard to MYC status.*

We chose to work with MDA-MB-231 cells for several reasons. From a practical standpoint, we have used MDA-MB-231 cells in previous publications. Further, MDA-MB-231 cells are a well-established TNBC cell line model. As suggested by the reviewer, we downloaded RNA-seq and gene amplification data from almost 70 breast cancer cell lines from DepMap. The Myc locus is not amplified in either MDA-MB-231 cells or the HCC70 cells added in the revision. From the expression data, we note that the expression of Myc and TXNIP are anti-correlated, which is the expectation from the results in our previous publication showing that Myc can suppress TXNIP expression. Second, this analysis revealed the MDA-MB-231 and the HCC70 cells express mid-range levels of both Myc and TXNIP mRNA (S1B Fig).

We have also analyzed TXNIP deletion in the immortal MB135 myoblast cell line, which do not have oncogenic Myc levels. TXNIP loss in this immortal, but not transformed cell line, also resulted in the upregulation of Myc-related gene signatures (S2G Fig), so oncogenic levels of Myc are not a strict requirement for TXNIP loss to increase Myc-dependent transcription.

*In Figure 6, comparing 231:TKO siMYC and 231 parental siMYC, are there pathways that are enriched in 231:TKO siMYC but not in the parental?  It might be useful to include a panel that outlines a few of these pathways, along with focusing on the change in enrichment in existing pathways in Figure 6E.*

There was excellent concordance between the Myc-dependent pathways regulated in the 231:TKO and parental 231 cells. The only pathway we identified that was enriched in the 231:TKO cells but not the parental 231 cells was Hallmark_Oxidative_Phosphorylation with a NES of -1.33 and FDR q-value of 0.173. We have mentioned this in the results (lines 536-538)

*The western blot in Figure S1E should be rerun so that the parental and TKO are on the same blot, and can be uniformly contrasted.*

Our apologies for the “frankenblot” in the original submission. The TXNIP and tubulin blots in the original figure were in fact from the same blot, but the shown lanes in the original were interspersed with a lane examining TXNIP and tubulin expression following MondoA knockout from the same MB135 cells. We omitted that lane for clarity in the original. We present a new blot from triplicate biological replicates in S2D Fig.

**Reviewer #3:**

*Overall these results are potentially interesting but preliminary and provide only limited additional information compared to the authors previously published work. There is essentially data from only one cell line, in vitro, without a specific mechanism, or demonstration that there is a causal role in tumorigenesis or even in change in metabolism. There is only limited effort to validate that other potential mechanisms have been considered.*

Below we address the reviewer’s concerns about 1) novelty, 2) limited analysis of a single cell line, 3) a causal role in metabolism, and 4) experiments to examine the role of TXNIP loss in in vivo xenograft experiments.

We feel strongly that the significance and novelty of our observation, even in the absence of a definitive mechanism, will be of interest to the general audience of PLoS Biology. We disagree with the reviewer’s comment that we extended “limited effort” to uncover the mechanism(s) that accounts for the increase in global Myc binding following TXNIP loss. Further, to imply otherwise devalues the effort expended to design and test the obvious and likely mechanistic connections between TXNIP and Myc. We have spent considerable effort testing whether TXNIP loss affects Myc:Max complexes, their nuclear:cytoplasmic distribution, stability and their association with known co-factors such as WDR5. We have also tested whether TXNIP loss changes global chromatin accessibility. We have included these data in the supplement (S9 and S10 Fig), so others might evaluate the rigor of our approaches. We are now pursuing more discovery-based approaches, such as novel Myc post-translational modifications and protein partnerships, and CRISPR-based screening that might explain why TXNIP loss has such a dramatic effect on Myc genomic binding.  We intend these experiments, which will take some time, to serve as the basis of a new follow-up manuscript.

*First, the experiments are essentially performed in one immortal human tumor derived cell line.*

In the original manuscript, we included an RNA-seq experiment from an immortal human myoblast cell, MB135 line in addition to the MDA-MB-231 cells. As with TXNIP loss in MDA-MB-231 cells, TXNIP loss in MB135 cells resulted in the increase in Myc gene signatures (S2G Fig). In the resubmission, we have included an additional TNBC cell line, HCC70 (S2A-C Fig). TXNIP loss in this cell line also results in increased Myc gene signatures (Fig 1E). In the original manuscript we also examined the association between TXNIP and Myc in 1904 breast cancers in the METABRIC database. In this analysis, we found that gene expression programs correlated with TXNIP were anticorrelated with Myc gene expression programs (Fig 1F). Our intention was not to imply that TXNIPs’ regulation of Myc is restricted to TNBC, as it certainly seems to extend across the different subtypes of breast cancer. Further, that TXNIP loss effects Myc transcriptional activity in the immortal myoblast cell lines suggests that transformation is not a prerequisite for TXNIP loss to drive Myc gene signatures. We have tried to clarify these points in the results (lines 113-127).

*Second, the results are only performed using in vitro data. And, there is no experimentation to see if any of the effects on gene expression or effects of the KO have any effects on actual tumor biology, invasive behavior, dependence or changes in metabolism.*

Our experiments show that TXNIP loss in MDA-MB-231,HCC70 and MB135 cells does not affect cell proliferation (S1D, S2C and S2F Fig) with the caveat we have only examined proliferation in serum-replete medium with 25mM glucose and supplemental glutamine. We provide new steady state metabolomics data, showing that TXNIP loss results in the upregulation of several progrowth metabolic pathways. Myc-knockdown experiments shows that their upregulation in TXNIP knockout cells is Myc-dependent (Fig 2E and 2F)

We have performed several orthotopic xenograft experiments with parental 231 and 231:TKO cells. We found that parental 231 and 231:TKO cells grow nearly identically in the mouse for about the first 5 weeks of the experiment at which time the growth rate of the 231:TKO-derived tumors slow dramatically. That the 231:TKO-derived tumors start to slow after about 5, suggests a potential involvement of the tumor vasculature, potentially controlling nutrient supply. On the surface, that 231:TKO cells can’t sustain tumor growth in orthotopic xenografts is counter-intuitive as TXNIP loss increases Myc-transcriptional programs. But several experiments from the Evan lab and others show that high Myc levels drive cell death, whereas lower levels support transformation and/or proliferation. Further, our 2015 paper with the Eisenman lab showed that MondoA loss is synthetically lethal with Myc overexpression. Many of our papers have established that TXNIP is the primary target of MondoA, so it is possible that TXNIP loss is also synthetically lethal with Myc overexpression. In our case, Myc need not be overexpressed as its genomic binding is increased by TXNIP loss. We are fascinated by this phenotype, but given the number of open questions, we have elected not to include this xenograft experiment in the manuscript. We have included this data below, so it can be evaluated by the reviewers.

*Third, the notion that MYC transcriptional effects are contextually dependent upon other gene products, metabolism, and/or transcriptional mechanisms has been well established by a multitude of investigators. The role of TXNIP and why this is novel beyond the authors prior work is not clear. Hence, the novelty and significance of the results needs to be clarified*.

Our previous PNAS publication only showed that Myc can repress TXNIP expression in MDA-MB-231 cells. Our current manuscript represents our effort to understand the consequences of TXNIP downregulation in TNBC. Our findings are novel in several ways: 1) we show that TXNIP loss increases Myc genomic binding without affecting its levels. Generally, many (potentially all) of the other influences on Myc transcriptional activity have been attributed to an increase in Myc levels. 2) our finding implies that Myc, through repression of TXNIP, drives a feedforward loop that reenforces its transcriptional activity. 3) Given that TXNIP expression is tightly regulated by both nutrient and progrowth signals, our results suggest that these signals coordinate, ultimately controlling, Myc transcriptional activity by regulating TXNIP expression. 4) our analysis is comprehensive, examining Myc genomic binding using ChIP-seq and determining Myc-dependence using knockdown approaches. 5) we put forth the concept that TXNIP loss expands the Myc-dependent transcriptome found in the parental 231 cells, rather than simply leading to an upregulation of the Myc targets in the parental 231 cells. This finding is similar, at least superficially, to others from the Eilers, Amati and Dang labs’ showing that increasing Myc levels allows it to occupy/invade the promoters and enhancers of “transformation” associated genes resulting in a qualitative shift in the Myc-dependent transcriptome. The difference here, of course, is that we see this qualitative shift in the Myc transcriptome in the absence of a change in Myc levels. We have tried to make these points in the abstract, introduction, and discussion as appropriate.

*Fourth, the presumption that there are no effects on MYC expression are based upon limited data, such as a single Western blot that is not quantified.*

We have now included a western blot from 3 biological replicates showing that Myc levels do not decrease following TXNIP deletion in MDA-MB-231 cells. This western blot is shown in S1C Fig and the quantification provided in Fig 1B. In the revision we have also added data from a TXNIP knockout of another TNBC cell line HCC70. Western blots from triplicate biological replicates and the subsequent quantification for HCC70 and MB135 cells are shown in S2A and S2D.

*Fifth, the mechanism proposed, that MYC promoter binding is globally enhanced as if MYC is overexpressed needs to be clarified. What is the mechanism? the authors think actually that a single gene KO always has the effect on MYC setting. Or only in this cell line? Or only in TNBC? How does this relate to the Levens and Young transcriptional enhancer model? Does this only explain "high" MYC then what is "high" versus physiologic (parental vs TKO)?*

In our response to reviewer 2 we outline the approaches we have taken to investigate mechanism. Our next major goal is to uncover the mechanism as this may reveal ways to regulate Myc in a therapeutic setting. As described above, our data clearly shows that that TXNIP loss can regulate Myc activity in more than just MDA-MB-231 cells. It will take additional experiments to know whether TXNIP loss “always” regulates Myc genomic binding and transcriptional programs. Given the complexities of different cell lineages and mutational landscapes across tumor cells, this seems unlikely. But nonetheless, a deeper analysis in additional cell lines and/or patient-derived models will help reveal the determinants that are required for TXNIP to regulate Myc genomic binding. In our response to reviewer 2, we discuss that our data is most consistent with Myc functioning as a site-specific transcriptional regulator rather than a global amplifier of gene expression. Further, and in our response to reviewer 2, is seems unlikely that TXNIP loss effects Myc transcriptional activity only in cells with high Myc levels as we see a similar increase in Myc gene signatures following TXNIP loss in immortal MB135 myoblast cells.
