# Supplementary material for: TXNIP loss expands Myc-dependent transcriptional programs by increasing Myc genomic binding
Source: PLoS Biol. 2023 Mar 17;21(3):e3001778. doi: 10.1371/journal.pbio.3001778 (PMC10058090; doi:10.1371/journal.pbio.3001778)
Supplement: Attachment — Submitted filename: PBIOLOGY-D-22-01559R2_RESPONSE_2-09-2023.docx [file pbio.3001778.s014.docx]

*Feb 10^th^, 2023*

Dear Richard,

We are so pleased that PLoSBiology is likely to publish our manuscript “TXNIP loss expands Myc-dependent transcriptional programs by increasing Myc genomic binding” (PBIOLOGY-D-22-01559R2). Below we address the remaining concerns point-by-point and we have uploaded the required supplemental data requested in (C) and original uncropped figures as requested in (F). We hope that with these changes that our manuscript is now acceptable for publication in PLoSBiology.

Sincerely, Don Ayer

*(A) We note that the new orthotopic xenograft data has been provided in the rebuttal but not in the manuscript. We ask that you please include this xenograft data in the paper and refer to the data in the manuscript text.*

We have included the xenograft data in two new data panels, Figure 2 G and H. We refer to this data in the results, discussion and methods sections. These changes should be easily identifiable in the “track changes” version of the manuscript.

*(B) In the Methods section, please include the full name of the IACUC/ethics committee that reviewed and approved the animal care and use protocol/permit/project license. Please also include an approval number.*

This information has been provided in the discussion as requested.

*(C) You may be aware of the PLOS Data Policy, which requires that all data be made available without restriction:* [*http://journals.plos.org/plosbiology/s/data-availability*](http://journals.plos.org/plosbiology/s/data-availability)*. For more information, please also see this editorial:* [*http://dx.doi.org/10.1371/journal.pbio.1001797*](http://dx.doi.org/10.1371/journal.pbio.1001797)

*Note that we do not require all raw data. Rather, we ask that all individual quantitative observations that underlie the data summarized in the figures and results of your paper be made available in one of the following forms:

-Supplementary files (e.g., excel). Please ensure that all data files are uploaded as 'Supporting Information' and are invariably referred to (in the manuscript, figure legends, and the Description field when uploading your files) using the following format verbatim: S1 Data, S2 Data, etc. Multiple panels of a single or even several figures can be included as multiple sheets in one excel file that is saved using exactly the following convention: S1_Data.xlsx (using an underscore).

-Deposition in a publicly available repository. Please also provide the accession code or a reviewer link so that we may view your data before publication.

Regardless of the method selected, please ensure that you provide the individual numerical values that underlie the summary data displayed in the following figure panels as they are essential for readers to assess your analysis and to reproduce it:

Figure 1B-C, 2B, 2D-F, 3C-D, 4A, 4C, 4D, 4F-G, 5D, 6C-G, S1A-B, S1D-E, S2B-C, S2E-F, S4A, S4C-D, S5C-E, S6A, S6C-D, S7E, S8B-C

NOTE: the numerical data provided should include all replicates AND the way in which the plotted mean and errors were derived (it should not present only the mean/average values).*

We have provided all of the requested in a supporting information file S1 Data, that has multiple datasheets in a Excel workbook.

*(D) Thank you for already depositing the RNA-seq, ChIP-seq and ATAC-seq data in the GEO. However, we note that this data is currently on hold and scheduled for release on July 17th 2023. We ask that you please ensure that this data is made publicly available before publication.*

GEO released this data on February 7^th^, 2023.

(E) Please also ensure that each of the relevant figure legends in your manuscript include information on *WHERE THE UNDERLYING DATA CAN BE FOUND*, and ensure your supplemental data file/s has a legend.

This information has been added to each figure legend as appropriate.

*(F) We require the original, uncropped and minimally adjusted images supporting all blot and gel results reported in the following figures:

Figure 1A, 2A, 4B, 4E, 6A, S1C, S2A, S2D, S10A-E*

*We will require these files before a manuscript can be accepted so please prepare and upload them now. Please carefully read our guidelines for how to prepare and upload this data:* [*https://journals.plos.org/plosbiology/s/figures#loc-blot-and-gel-reporting-requirements*](https://journals.plos.org/plosbiology/s/figures#loc-blot-and-gel-reporting-requirements)

We have uploaded the original figures as S1_raw_images.pdf

(G) Please ensure that your Data Statement in the submission system accurately describes where your data can be found and is in final format, as it will be published as written there.
